# Genomic Insights Into Local Adaptation Across Heterogeneous Understory Habitats and Climate Change Vulnerability

**DOI:** 10.1111/mec.70068

**Published:** 2025-08-18

**Authors:** Nan Lin, Yakun Wang, Xiankun Wang, Yuxuan He, Xianhan Huang, Qun Liu, Hengchang Wang, Tao Deng

**Affiliations:** ^1^ State Key Laboratory of Plant Diversity and Specialty Crops, Kunming Institute of Botany Chinese Academy of Sciences Kunming Yunnan China; ^2^ College of Life Science Henan Agricultural University Zhengzhou Henan China; ^3^ Henan Engineering Research Center for Osmanthus Germplasm Innovation and Resource Utilization Henan Agricultural University Zhengzhou Henan China; ^4^ College of Landscape Architecture and Art Henan Agricultural University Zhengzhou Henan China; ^5^ CAS Key Laboratory of Plant Germplasm Enhancement and Specialty Agriculture, Wuhan Botanical Garden Chinese Academy of Sciences Wuhan Hubei China

**Keywords:** adaptive evolution, climate change, genomic offsets, genomic variation, genotype–environment associations

## Abstract

Understanding adaptive evolution and survival risks in understory herbs is crucial for the effective conservation of biodiversity. How environmental gradients shape species local adaptation patterns is not well understood, nor is how populations of understory herbs respond to a changing climate. In this study, we conducted population genomic analyses of *Adenocaulon himalaicum* (Asteraceae) with a pan‐East Asian distribution, representing a good model for dominant understory herbs to elucidate adaptation mechanisms in heterogeneous forest ecosystems. Based on 34,398 putatively neutral single nucleotide polymorphisms (SNPs) across 27 populations, we identified three genetic lineages accompanied by high levels of genetic differentiation between populations. Our isolation by environment results (IBE) indicated a significant effect of environmental gradients on genomic variation of *A. himalaicum* (*r* = 0.18, *p* = 0.03). To decompose the relative contributions of climate, geography and population structure in explaining genetic variance, our partial RDA found that the prominent contribution of environmental effects (climatic and soil variables) explained 29% and 36% of the neutral and adaptive genetic variation, respectively. Using two genotype–environment association (GEA) methods, we identified 13 SNPs as candidates for core climate‐related adaptation loci, with two of these loci further validated by qRT‐PCR experiments. Projections of spatiotemporal genomic vulnerability under different future climate scenarios revealed that populations in the southeastern edge of the Himalayas, near the Sichuan Basin, the southernmost region of Northeast China and the northern Korean Peninsula, as well as northern Japan, were identified as the most vulnerable and should be prioritised for conservation. Therefore, our current study provides the genomic foundations for conservation and management strategies to elucidate how these understory herbs cope with future climate changes.

## 1 Introduction

Understory herbaceous plants are one of the most important components of East Asian temperate vegetation, playing a crucial role in the recruitment of trees and soil conservation (Deng et al. 2023; McLachlan and Bazely 2001). However, global climate change threatens biodiversity, altering the distributions of species and their composition in understory forest vegetation ecosystems (Feeley et al. 2020; Harrison et al. 2015; Lippmann et al. 2019). Herbaceous plants are often widely distributed and can adapt to a variety of habitats, especially in open grasslands, wastelands and mountain slopes, while being subject to significant human disturbance (Sheremet'ev and Gamalei 2012). At present, severe fragmentation of forest habitats in East Asia, accompanied by local genetic erosion and limited dispersal capacity, makes understory herbs particularly vulnerable to climate change (Tian et al. 2023; Van Daele et al. 2022).

Local adaptation is one of the most effective ways for plants to cope with climate changes (Fitzpatrick and Keller 2015; Waldvogel et al. 2020), and extreme climatic factors such as temperature and precipitation have been shown to drive plant adaptation in both forest trees and crops (e.g., Cao et al. 2020; Lazic et al. 2024; Sang et al. 2022). For instance, genomic analyses in *Dasiphora fruticosa*, *Prunus mira* and *Salix brachista* revealed that alpine plants have accumulated a number of genetic variants associated with high altitude, low oxygen and intense solar radiation (Chen et al. 2019; Wang et al. 2021; Yang et al. 2024). Drought‐resistant genes are commonly identified under positive selection in desert plants, such as *Gymnocarpos przewalskii* and *Sophora moorcroftiana* (Fu et al. 2024; Yin et al. 2023). Likewise, several studies have demonstrated that halophytic plants exhibit pronounced signatures of selection on osmo‐homeostatic regulators for maintaining cytoplasmic ion homeostasis (Cui et al. 2024; He et al. 2022; Wang et al. 2017). While studies using large‐scale sample plots have significantly enhanced our knowledge of composition and distribution patterns of understory herb communities (Dormann and Woodin 2002; Landuyt et al. 2019; Møller et al. 2023), there remain significant gaps in understanding the genetic basis of local adaptation and evolutionary fate of understory herbs under future climate scenarios (Liao et al. 2022; Niu et al. 2024). Therefore, deciphering genomic signatures of adaptive evolution and quantifying survival risks across heterogeneous landscapes are vital for the development of conservation plans for understory vegetation in East Asia (Van Daele et al. 2022). Although empirical studies have largely confirmed the utility of genomic approaches in deciphering underlying climate‐driven adaptation in trees (e.g., Cao et al. 2020; Lazic et al. 2024; Sang et al. 2022), a limited number of studies focus on how understory herbs respond to future climate changes. Thermophilic xerophytes in particular may exhibit inherent advantages of genomic adaptations conferring resilience to the projected climate warming of the next century (Landuyt et al. 2018). Critical knowledge gaps remain regarding understory herbaceous species in cold‐mesic ecosystems. However, in empirical systems, phenological and physiological lags decouple plant responses to changes in climate, thus making it highly challenging to understand whether understory herbs occupying cold and mesic habitats will be more vulnerable under future warming scenarios (Ahrens et al. 2019; Alexander et al. 2017; Duchenne et al. 2021).

Recent developments in landscape genomics are enabling unprecedented insights into the evolutionary processes and molecular basis of adaptation (Feng et al. 2024; Izaguirre‐Toriz et al. 2024; Waldvogel et al. 2020; Yuan et al. 2023). In contrast to long‐term fieldwork and common garden experiments, genotype–environment association (GEA) approaches integrate the ecological and evolutionary methods to identify loci involved in local adaptation (Aguirre‐Liguori et al. 2019; VanWallendael et al. 2019). Multivariate analyses effectively elucidate the relationships between genotypes and environmental factors, thereby revealing the relative contributions of environmental variables in shaping genetic variation (Rellstab et al. 2016). Furthermore, through analyses of GEA, we can identify candidate loci that play crucial roles in adaptation to specific climate conditions. Based on the candidate genes involved in adaptive genetic variation, quantitatively predicting the genetic mismatch, thereby assessing the amount of change in the genetic composition of a population for adapting to future environmental conditions, is possible (Dauphin et al. 2020; Gougherty et al. 2021). Accordingly, populations with higher genetic mismatch (i.e., genetic offsets) are predicted to be more vulnerable and exhibit high extinction risks under future climate changes (Fitzpatrick and Keller 2015). This approach has effectively highlighted adaptive evolution and evolutionary potentials under climate changes for various trees (e.g., Ruegg et al. 2018; Sang et al. 2022; Yuan et al. 2023), but its application in characterising genome‐wide evolutionary risks for understory herbaceous plants remains largely unexplored. The driving mechanisms of climatic factors on trees and understory herbs exhibit substantial differences, primarily reflected in phenological responses, microenvironmental sensitivity and the temporal scales of adaptation (Murphy et al. 2016; Radhamoni et al. 2023). Tree populations, characterised by their long lifespans, predominantly reflect broad‐scale climatic gradients, whereas understory herbaceous species demonstrate greater sensitivity to microclimatic buffering effects (Deng et al. 2025; Spicer et al. 2020). In contrast to trees, which predominantly exhibit pollen‐mediated gene flow that maintains panmictic populations at landscape scales, understory herbs often develop fine‐scale genetic structuring due to limited seed dispersal or clonal propagation (Petit et al. 2005). Existing genomic studies of understory herbs (e.g., *Ardisia*, *Impatiens*, *Trillium*) have primarily investigated genetic diversity and pollination syndromes (Gonzales and Hamrick 2005; Xue et al. 2025; Zeng et al. 2012), while few studies explicitly addressed climate adaptation or assessed the risk of maladaptation under future climate change, hindering the formulation of precise conservation strategies for understory herbs. Thus, applying forest tree‐focused landscape genomic frameworks to quantify the evolutionary potential of an understory herb is not only essential to understand the molecular mechanisms needed to respond to altered environmental conditions; it can also contribute to the preservation and sustainable management of global vegetation biodiversity (Waldvogel et al. 2020).

*Adenocaulon himalaicum* Hook. (Asteraceae) is an ecologically and medicinally important herb species widely distributed across East Asia (Chen and Hind 2011). Unlike most Asteraceae species that rely on wind‐driven seed dispersal via pappus structures, *A*. *himalaicum* has evolved a distinct dispersal strategy through glandular achenes. This propagule morphology represents an evolutionary synapomorphy among understory herbs, sometimes facilitating long‐distance dispersal through avian and anthropogenic agents spanning several to hundreds of kilometres (Bittmann 1990). *A. himalaicum* thrives in diverse habitats, including riverbanks, ravines, mountain slopes and subalpine zones, with an elevational range spanning from 500 to over 4000 m. This species prefers humid (annual precipitation: ca. 1000 mm) and cold (annual mean temperature: ca. 9°C) environments of temperate broad‐leaved and coniferous forests. The Asian monsoon plays a crucial role in shaping the geographic distribution pattern of *A. himalaicum*, suggesting local adaptation to divergent climatic conditions across different populations (Deng et al. 2018). Therefore, *A. himalaicum* serves as an ideal representative to explore adaptive evolution and potential genomic vulnerability in understory herbs preadapted to cold and humid conditions in East Asia.

In this study, we sequenced 221 individuals of *A. himalaicum* from 27 populations covering its entire distribution in East Asia. Based on restriction site‐associated DNA sequencing (RAD‐seq) data, we integrated population genomic, landscape genomic and molecular biology methods by genomic‐level SNPs of *A. himalaicum* to ask the following unresolved questions: (1) Are the patterns of genetic diversity within *A. himalaicum* associated with landscape heterogeneity? (2) What is the genomic basis of local adaptation among *A. himalaicum* populations and the potential genetic pathways related to the adaptive process? (3) What is the spatial distribution of genomic vulnerability and are the populations occupying more moist and cold conditions more vulnerable to future climate changes?

## 2 Materials and Methods

### 2.1 Sample Collection, DNA Extraction and Sequencing

**FIGURE 1:**
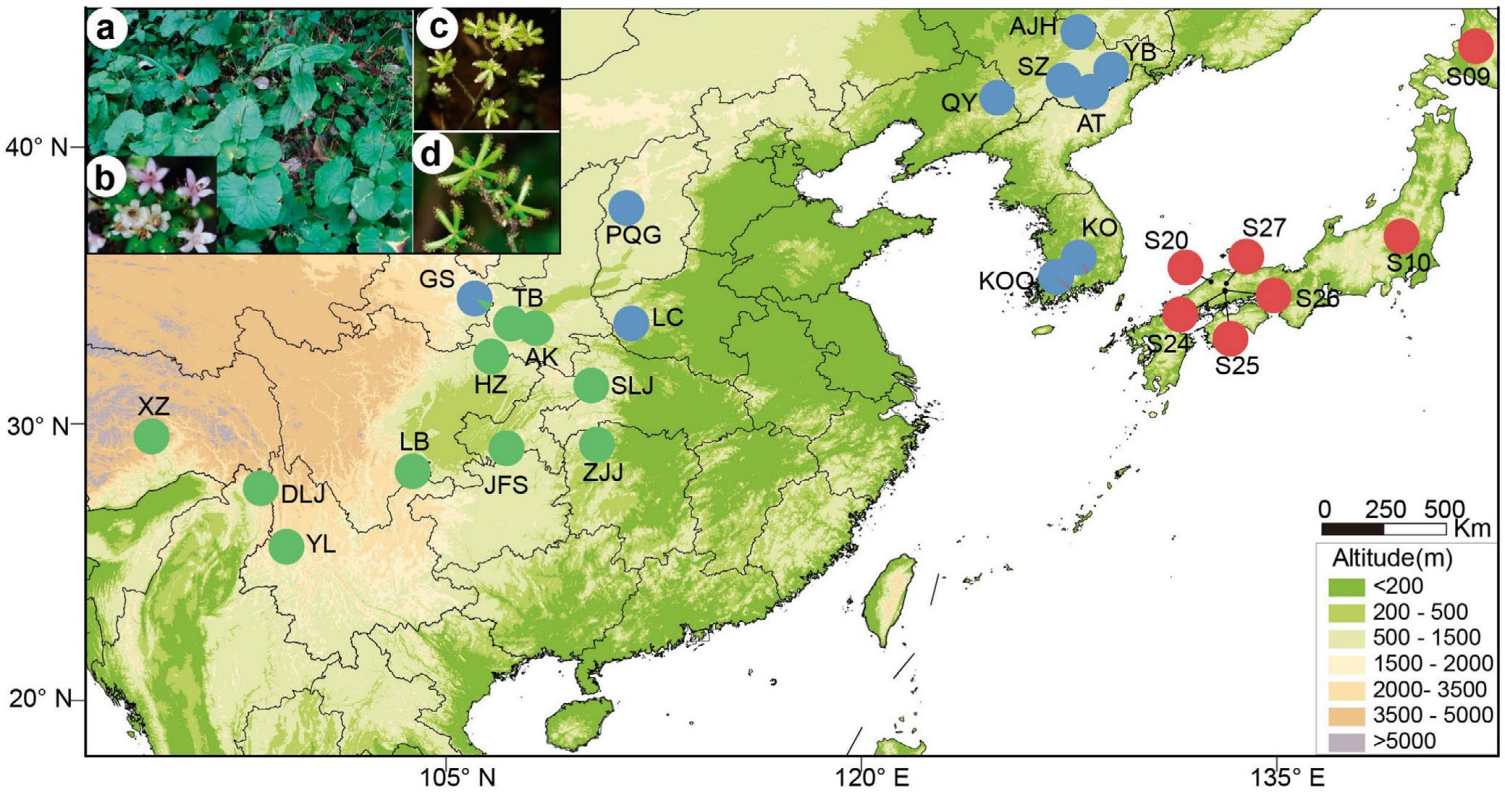
Geographic distribution of 27 natural populations of *A. himalaicum*, where colours of pies correspond to the ancestry composition of each population for *K* = 3 inferred using Admixture. The plant, flower and fruit are shown in the upper left corner.

In total, 221 individuals were collected from 27 populations of *A. himalaicum* across its geographic distribution in East Asia, including northern and southern China, the Korean Peninsula and Japan (Table S1; Figure 1). Within each population, 2–17 individuals were sampled at least 50 m apart to maximise genetic diversity of the samples. Fresh leaves from individuals were dried and preserved in silica gel. Genomic DNA was extracted using a modified cetyltrimethylammonium bromide (CTAB) method with further measures of DNA quality control according to Doyle (1991). DNA degradation and contamination were checked on 1% agarose gels. DNA purity and concentration were checked using a NanoPhotometer spectrophotometer (Implen, CA, USA) and a DNA Assay Kit in a Qubit 2.0 Fluorometer (Life Technologies, CA, USA), respectively. RAD library construction and sequencing were performed by Novagene (Beijing, China). Briefly, the genomic DNA (~1 μg) was digested using the restriction enzyme *EcoRI* (5′‐GAATTC‐3′, New England Biolabs (NEB)), and a P1 adapter was ligated with the compatible ends of the fragments. The adapter‐ligated fragments were subsequently pooled, randomly sheared and size‐selected. The DNA was then ligated to a modified P2 adapter. Finally, DNA fragments of 300–500 bp were collected for library construction. The libraries were sequenced on an Illumina NovaSeq 6000 platform with 150 bp paired‐end reads. Four sequencing lanes were used for the initial sequencing of 221 individuals, and another two lanes were used to increase the coverage of samples.

### 2.2 Raw Data Processing and SNP Calling

Sequencing quality of the raw reads was initially checked with FastQC to measure the quality scores per base, sequence length consistency, and unknown base (*N*) ratios for downstream analyses (Andrews 2010). The paired‐end reads were demultiplexed and quality filtered with the ‘process_radtags’ module, and PCR duplicates were identified and removed from each library using the ‘clone_filter’ model from Stacks v2.41 (Catchen et al. 2013). Due to a lack of an available reference genome for *A. himalaicum*, the ‘denovo_map.pl’ module of Stacks v2.41 was used for SNP calling (Catchen et al. 2013). Accordingly, we used ‘ustacks’ for assembly by clustering reads to tags identified with the minimum number of raw reads to create a stack (*m* = 4), allowing for two mismatches within stacks of the same individual (*M* = 2). Then ‘cstacks’ was used to build a catalogue of consensus loci by clustering tags from each individual as well as all merged alleles, with the number of mismatches allowed between loci to be no more than two. The aligned tags from each individual were matched to the catalogues by ‘sstacks’. Finally, the ‘populations’ model was used to filter the loci according to loci with minor allele frequencies < 0.05 and a maximum observed heterozygosity set to 0.5 to reduce the potential paralogs (Roesti et al. 2012). Only the first SNP per locus in the final analysis was kept to avoid linkage bias, and polymorphic loci present in at least 85% of the individuals and at least 19 populations were retained. PGDSpider v2.2.1 and VCFtools v0.1.16 were then used to generate input files for downstream analyses (Danecek et al. 2011; Lischer and Excoffier 2012).

### 2.3 Identification of Neutral SNPs for Genetic Analyses

To minimise false positives, three *F*_ST_‐based methods were used to identify putative outlier SNPs with high levels of differentiation among all 27 populations (Foll and Gaggiotti 2008). We first performed BayeScan v2.1 to identify outlier loci using a Bayesian method based on a logistic regression model (Foll and Gaggiotti 2008). BayeScan was implemented using a prior model of 1000, with a burn‐in of 200,000 and 20 pilot runs of 10,000 iterations, and the SNPs with a false discovery rate (FDR) value below 0.05 were considered as *F*_ST_ outliers. PCADAPT v4.2.3 was then used to detect putatively differentiated genetic loci (Privé et al. 2020) by identifying SNPs that significantly deviated from the neutral background structure along the principal components with an FDR‐adjusted *p*‐value threshold of 0.05. In addition, ‘fdist2’ in Lositan v1.0 was performed to evaluate *F*_ST_ outliers according to the expected neutral relationship between *F*_ST_ and the expected heterozygosity across all loci (Beaumont and Nichols 1996). fdist2 was run under an infinite allele model with 1,000,000 simulations and the ‘neutral mean *F*_ST_’, while putative outliers were identified from the top 1% of loci with an FDR less than 0.05 to control for the method's inherent susceptibility to false positives. Finally, an SNP was considered to be putatively under divergent selection if all three of the above methods identified it as an outlier across the 27 populations. Based on the results of these three analyses, we divided all SNPs into two datasets: putatively neutral and putative *F*_ST_ outliers. The putatively neutral SNPs were then used for the following genetic diversity and genetic differentiation analyses. Population genetic statistics, including nucleotide diversity (Pi), observed heterozygosity (*H*_O_), expected heterozygosity (*H*_E_), pairwise population *F*_ST_ and fixation index (*F*_IS_) for each *A. himalaicum* population, were estimated using the ‘population’ model in Stacks v2.41 (Catchen et al. 2013). Admixture v1.23 was employed to infer the most likely number of ancestral populations using the neutral SNPs (Alexander et al. 2009). To determine the optimal clustering of populations, the cross‐validation cluster error value was calculated with *K* from 1 to 15, while the *K* with the lowest cross‐validation error was considered as the optimal value. Population divergence was also analysed by principal component analysis (PCA) using genome‐wide complex trait analysis (GCTA) v1.26 (Yang et al. 2011). A maximum likelihood (ML) phylogenetic tree was constructed to explore evolutionary relationships using IQTree v2.0.3 under an optimal substitution model determined from the ModelFinder program (Minh et al. 2020).

### 2.4 Inference of Demographic History

To investigate the demographic history of the 27 populations within *A. himalaicum*, we used the SFS‐based composite likelihood demographic modelling method implemented in FASTSIMCOAL v2.8 (Excoffier et al. 2013). Based on our phylogeny from the chloroplast dataset (unpublished), the individuals were divided into three lineages, with the southern populations diverging first, followed by the split between the northern populations and the Japan populations. Thus, our demographic models aimed at exploring the changes in effective population sizes during divergence (i.e., expansion, contraction, expansion‐contraction and contraction‐expansion) and the presence/absence of gene flow for three genetic groups. To avoid the effects of missing data on the inference of population history, we performed coalescent simulations on a high‐quality data matrix (1524 SNPs with less than 10% missing data across all individuals) and calculated the folded SFS using easySFS (https://github.com/isaacovercast/easySFS). We maximised the likelihood of the observed SFS under a total of 10 gene flow scenarios and 6 demographic scenarios to test the presence/absence of gene flow and the changes in effective population sizes, respectively (Figures S1 and S2). The best‐fitting demographic model was identified on the basis of its Akaike's information criterion (AIC) score. We set the mutation rate to 7 × 10^−9^ per site per generation following *Arabidopsis thaliana* (Ossowski et al. 2010) and set the generation time as one year based on field observations. We first ran 50 independent iterations with 100,000 coalescent simulations and 40 optimisation cycles and obtained point estimates of the demographic parameters of the model based on the highest maximum composite likelihood. Then, to evaluate the uncertainty of the data, 95% confidence intervals (CIs) of the parameters were obtained using a parametric bootstrapping procedure, creating 100 pseudo‐observed SFS using the point estimates of the parameters to repeat the estimations.

### 2.5 Spatial Structure and Environmental Factors

The 19 bioclimatic variables under current conditions (1970–2000) were extracted from WorldClim v2.1 raster layers at 30 s resolution (~1 km^2^; Table S2; Hijmans et al. 2005). Five soil factors were extracted from the Harmonised World Soil Database v1.21 at 30 s resolution (~1 km^2^; Table S2; Nachtergaele et al. 2012). In addition, the Moran eigenvector maps (MEMs) are regarded as independent vectors that summarise the spatial structure across population locations (Borcard and Legendre 2002). Therefore, we calculated MEMs as spatial eigenvector predictors using the ‘adespatial v0.3‐28’ package (Dray et al. 2018), and three significant spatial factors (MEM1, MEM2 and MEM3) were retained for further analysis. Gradient forest (GF) analysis was conducted across all populations and three genetic groups to assess the contribution of the 24 environmental variables, spatial eigenvectors and genetic structure to patterns of genetic variation using the ‘gradientForest v0.1‐17’ package (Ellis et al. 2012) in R v4.3.2. The GF modelling used a non‐parametric machine learning regression algorithm tree to explore non‐linear associations of the environmental and allelic variables (Fitzpatrick and Keller 2015). To avoid multicollinearity, a Pearson correlation coefficient (|*r*| > 0.75) combined with the GF ranked accuracy importance among all the variables was employed to remove highly correlated variables. Finally, a total of ten environmental variables (five climate variables: mean diurnal range of temperature (bio 2), isothermality (bio 3), maximum temperature of warmest month (bio 5), precipitation seasonality (bio15), precipitation of warmest quarter (bio18)), along with five soil variables (cation exchange capacity of soil (CEC), organic carbon density of soil (OCD), the content of sand (sand), taxonomic class in the world reference base system (TAXNWRB) and pH of soil (PH_H2O)), three proxies of neutral genetic structure (PC1, PC2 and PC3), and spatial factors (MEM1, MEM2 and MEM3) were retained as final variables for subsequent analyses.

### 2.6 Association Between Genetic Variation and Environmental Gradients

To investigate the relative role of geography and environment gradients in shaping spatial neutral genetic variation, a Mantel test was conducted for isolation by distance (IBD) and isolation by environment (IBE) across all 27 populations using the ‘vegan’ package in R v4.3.2 with 999 permutations (Oksanen 2015). The geographical distances between populations were calculated using the ‘geosphere v1.5‐20’ package (Hijmans 2016). For the environmental distances between populations, a PCA was performed with the environmental variables across all 27 populations, and the first two principal components (Clim_PC1 and Clim_PC2) values were used to calculate a pairwise distance matrix (Pluess et al. 2016). To decompose the contribution of neutral population structure for the purpose of explaining genetic variation, we performed a correction for population structure in RDA according to Capblancq and Forester (2021). Briefly, we used three proxies of neutral genetic structure (population scores along the first three axes of a genetic PCA from neutral SNPs) to remove the climatic dissimilarity confounded with population structure. The three significant spatial factors (MEM1, MEM2 and MEM3) were calculated, and the 10 independent climatic and soil variables (see above) were used as environmental variables. Partial redundancy analyses (pRDAs) were also used to detect linear relationships between genetic variation and multivariate environmental gradients (i.e., environmental variables, population structure and spatial factors) across all 27 populations and three genetic groups using the ‘vegan’ v2.7‐1 package (Oksanen 2015) with 999 permutations.

### 2.7 Screening of Genome–Environment Association (GEA) Loci

Two different approaches were combined to identify environment‐associated loci based on non‐*F*_ST_ outlier SNPs across 27 *A. himalaicum* populations. First, we used a univariate latent‐factor linear mixed model (LFMM) implemented in the ‘lfmm v1.1’ package (Caye et al. 2019) to search for associations between allele genotype and 10 environmental variables, with three latent factors to account for population structure based on Admixture v1.23 results (Frichot and François 2015). We ran each environmental variable independently, and the Benjamini–Hochberg FDR method was used to correct for multiple testing (Benjamini and Hochberg 1995). All SNPs showing significant association (FDR < 0.05) with any of the variables were considered as GEA outliers. In addition, we ran redundancy analysis (RDA) to explore genetic variants related to multivariate environmental axes. Significantly associated environment loci were defined as those having loadings in the tails of the distribution using a standard deviation cutoff of three along one or more RDA axes (Capblancq and Forester 2021). The intersecting loci of the two approaches were used as final core GEA loci, which we analysed across the 27 populations and within the three genetic groups. To identify locally adaptive genetic variants with geographic patterns across the entire distribution range, we further annotated these core GEA loci using transcriptomes of *A. himalaicum* from the National Center for Biotechnology Information (accession number: SRR12917369). Transcriptomes of *A. himalaicum* were *de novo* assembled and annotated using Trinity v2.13.2 and TransDecoder v5.3.0 (Haas et al. 2013; Grabherr et al. 2011), respectively. The consensus sequences of the candidate GEA locus were annotated through a BLASTN analysis (E‐value cutoff = 1 × 10^−5^) against the nucleotide database of the assembled transcripts. Subsequent function annotations for the identified transcripts were performed using the *A. thaliana* TAIR database (https://www.arabidopsis.org/).

### 2.8 Stress Treatment and Expression Analysis by qRT‐PCR

Thirty wild individuals from the mid‐latitude population LC were grown under 25°C and a photoperiod (16/8 h light/dark cycle). Plants were subjected to routine management, including watering and fertilisation. After 10 days of culture, 12 plants with uniform and robust growth were selected for stress treatment. To investigate the potential function of representative candidate GEA loci, we simulated cold and drought treatments to test the expression levels of the selected genes under abiotic treatments at 9:00 am local time. For the cold stress treatment, plants with three biological replicates were exposed to low temperatures of 4°C for 0, 2, 4, 8 and 12 h. For the drought stress treatment, plants were watered with polyethylene glycol (PEG‐6000; 20 g·L^−1^) for 0, 2, 4, 8 and 12 h. For each plant, leaf tissue was collected at each time point from the same place, immediately frozen in liquid nitrogen, and stored at −80°C for RNA extraction. To test the gene expression levels by quantitative reverse transcription PCR (qRT‐PCR), RNA was extracted from pooled leaf materials using a Plant RNA extraction kit (Biofit, Chengdu, China) and visualised on 1% agarose gels. The RNA purity (OD260/280 and OD260/230) and integrity were measured using a NanoPhotometer spectrophotometer (Implen, CA, USA). The HiScript II RT SuperMix for qPCR kit (+gDNA wiper) (Vazyme, Nanjing, China) was used to obtain cDNA and qPCR was performed using the Taq Pro Universal SYBR qPCR Master Mix (Vazyme, Nanjing, China) reaction system on the CFX96 Real‐Time detection system (Bio‐Rad). Each experiment was performed with three technical replicates, and the *ACT1* gene was used as the endogenous control for data analysis.

### 2.9 Genomic Vulnerability Under Future Climate Change

For each population site of *A. himalaicum*, 19 future environmental variables (including 2041–2060 and 2061–2080) were downloaded from the MIROC6 model at a resolution of 2.5 min. Both low and high emission scenarios (shared socioeconomic pathways (SSPs): SSP126 and SSP585) were used to evaluate the genomic offsets to future climate change. To predict genetic vulnerability under future climate conditions across 27 populations, we first performed Gradientforest (GF) analysis to predict the genomic composition of each grid point across the range of *A. himalaicum* (Ellis et al. 2012). The GF model was tested using 500 regressions per SNP, and we computed the Euclidean distance between current and future genetic compositions (Fitzpatrick and Keller 2015). The results for different future scenarios were visualised to display their spatial distribution with ArcGIS v10.2. Furthermore, we integrated migration to predict potential maladaptation to future climate change and calculated three different formulations of genetic offset: the local, forward and reverse offsets for each future scenario (Gougherty et al. 2021). The local offset was used to predict changes in allele frequencies necessary to respond to future climates. Forward genetic offset was calculated as the minimum predicted offset, assuming populations have unlimited migration ability. Specifically, we investigated the forward offset changes when the maximum migration distance was set to a gradient of 50, 100, 200, 500 km and unlimited to encompass both biologically plausible dispersal capacities and extreme scenarios in *A. himalaicum*. Reverse offset is similar to the forward offset but was calculated from the future climate to the current climate, representing the possibility that populations in the current range would be preadapted to a particular location in the future. Reverse offset was quantified by assessing the minimum genetic offset between hypothetical future populations within the current geographic range and extant populations under contemporary climate conditions (Gougherty et al. 2021). Furthermore, we mapped these three metrics as the red, green, and blue bands of an RGB image to visualise local, forward and reverse offsets simultaneously.

## 3 Results

### 3.1 Characterisation of Sequencing Data and Neutral SNPs

After quality control for raw reads in *A. himalaicum* populations, the minimum and maximum number of sequencing reads per population were 5,937,684 and 63,296,772, respectively (Figure S3). The average depth was 8.08× for stacks clustering in each individual, ranging from 5.37× to 21.80× (Figure S4). After stringent filtering, a total of 34,411 SNPs were retained for the total of 27 populations in *A. himalaicum* for subsequent analysis (Figure S4). Among these SNPs, 1161 *F*_ST_ outlier SNPs were detected from BayeScan, with 10,512 and 3677 *F*_ST_ outlier SNPs identified by fdist2 and PCADAPT, respectively (Figure S5). The 13 *F*_ST_ outlier SNPs identified by all three methods were retained for further analyses as *F*_ST_ outlier SNPs (Figure S5). Finally, 34,398 SNPs were defined as the neutral SNPs dataset.

### 3.2 Analysis of Population Structure and Genetic Diversity

According to our PCA and phylogenetic results, the 27 populations were divided into three groups, which geographically correspond to southern China (hereafter named group SC), southern Northeast China and the Korean Peninsula (hereafter named group NK) and Japan (hereafter named group JA), respectively (Figures 1 and S6). Admixture results were highly consistent with the PCA and phylogeny when the optimal number of ancestral populations was equal to three. In the PCA results, the first two axes correspond to 35.27% and 11.07% of the total genetic variation (Figure S6). The resulting PCA plots resemble a geographical map of the *A. himalaicum* distribution, with the first axis of PC1 populations arranged along a south–north axis at 35° N and the second axis of PC2 mainly separating the southern populations from other regions (Figures 1 and S6). Furthermore, genetic differentiation (*F*_ST_) supports a high genetic differentiation between the three lineages (Figure S7). Among the 27 populations, the highest genetic differentiation and lowest genetic differentiation were 0.925 (population LC vs. population DLJ) and 0.150 (population SZ vs. population AJH), respectively (Figure S8). Nucleotide diversity (π) ranged from 0.010 (S09) to 0.079 (AJH) with an average value of 0.036 (Table S1). The NK population had the highest genetic diversity (0.043), and the JA population had the lowest genetic diversity (0.026). Population genetic diversity (*r* = 0.52, *p* < 0.05) and the expected heterozygosity increased with latitude (r = 0.53, *p* < 0.05; Figure S9).

### 3.3 Demographic History Inference

**FIGURE 2:**
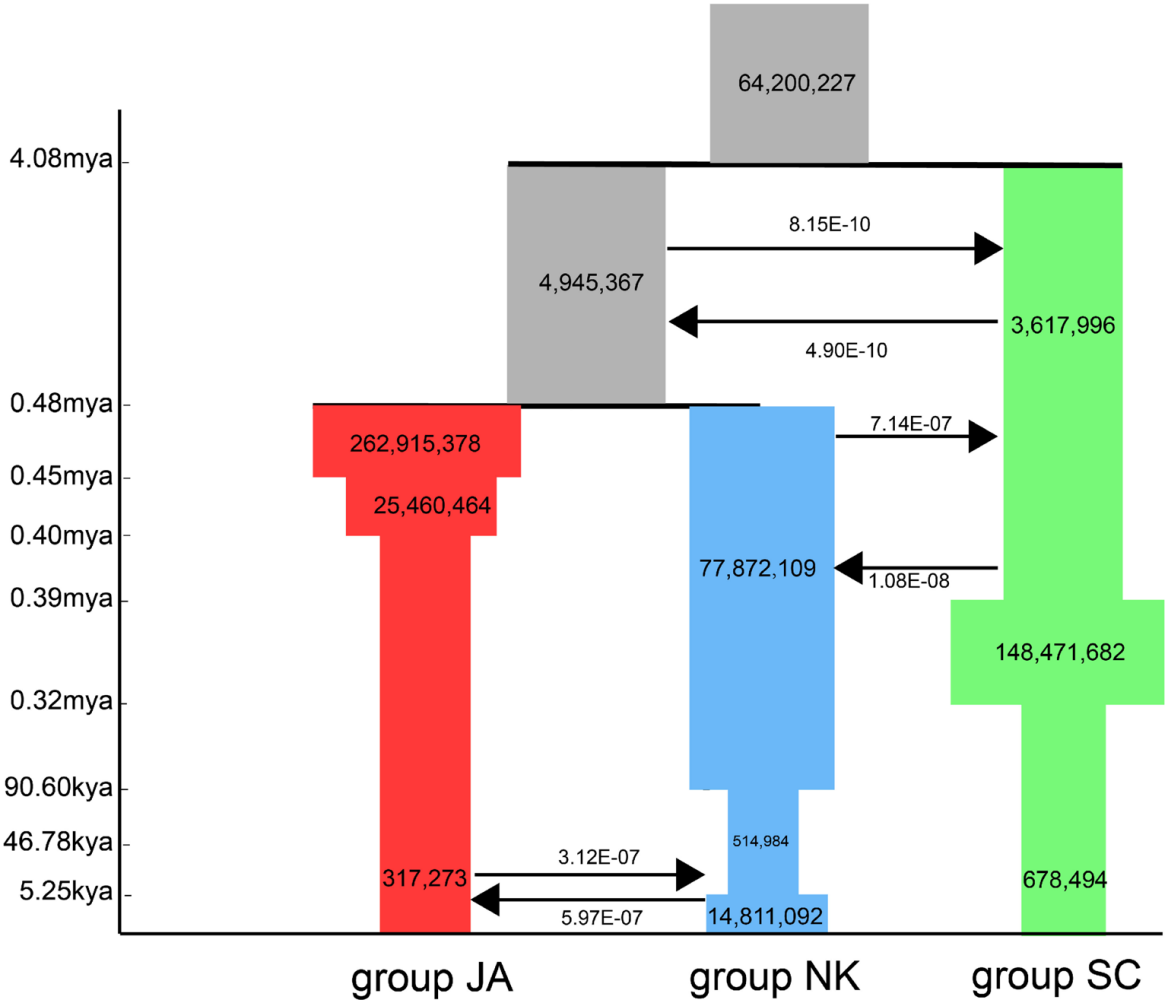
The demographic history of *A. himalaicum* inferred by FASTSIMCOAL 2 from the best‐fit mode. Red, blue and green columns correspond to the historical changes in effective population size (*Ne*) through time of three genetic groups, with the ancestral population shown in grey. The area and width of the columns are proportional to the relative effective population size. Gene flow events are marked by arrows, and the numbers on the vertical axis indicate the estimated time of population historical events.

According to the best‐fit FASTSIMCOAL model for *A. himalaicum* (Figure 2, Table S3), the southern China lineages (group SC) diverged from their common ancestor during the Pleistocene to Pliocene, c. 4.08 Mya, and the Northern Korean (group NK) and Japan lineages (group JA) split approximately 0.48 Mya. Based on the ten demographic scenarios of gene flow between the three groups of *A. himalaicum* (Figure S1), the simulated results detected ancient gene flow between SC and the common ancestor of NK and JA (Figure 2). Subsequently, a low‐level bi‐directional gene flow can be detected between NK and SC and between NK and JA after their divergence. According to the six demographic scenarios of effective population changes of *A. himalaicum* (Figure S2), the best‐fit FASTSIMCOAL model confirms divergent expansion and contraction demographic scenarios across the three genetic groups (Figure 2). For SC, the best‐fit model supports an expansion‐contraction model, the populations of which experienced an initial expansion 0.39 Mya, followed by a long‐term reduction in population size (c. 200‐fold) from 0.32 Mya. In contrast, the populations in NK follow a contraction‐expansion model from 90.6 kya and 5.25 kya, respectively. For JA, the populations experienced a constant decrease beginning from 0.45 Mya.

### 3.4 Associations Between Environmental Variables and Genetic Variation

**FIGURE 3:**
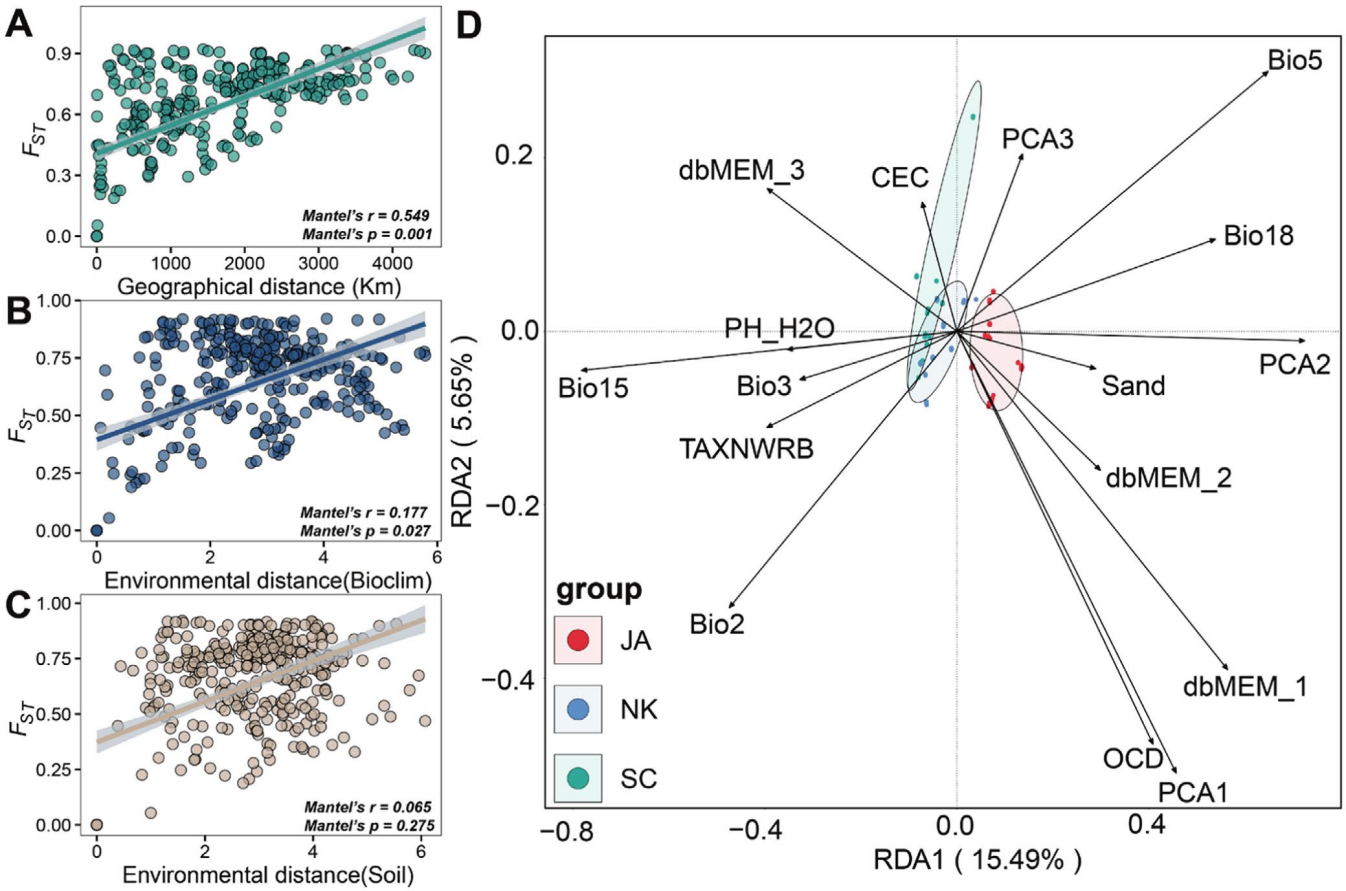
Mantel tests of isolation by distance and isolation by environment analyses for 27 populations based on neutral SNPs. (A) Correlation of mean pairwise geographic distance versus mean pairwise *F*_ST_; (B) Correlation of mean pairwise environmental distance of bioclimatic factors versus mean pairwise *F*_ST_; and (C) pairwise environmental distance of soil factors versus mean pairwise *F*_ST_. The shadow around linear regression denotes the 95% confidence interval. (D) RDA analysis for multivariate SNPs and environment associations.

The Mantel tests support a significant positive correlation between population geographic distances and genetic distances (*r* = 0.549, *p* = 0.001; Figure 3A). At the same time, a significant correlation between climatic distances and genetic distances (*r* = 0.177, *p* = 0.027, Figure 3B) was also detected. In the partial RDA, the total contribution of geography, genetic structure and environmental variables (including climate and soil variables) significantly explains 56% neutral genetic variance across all populations (Table S4), while accounting for 82%, 73% and 71% neutral genetic variance in SC, JA and NK, respectively. The effects of climate and soil explain the highest neutral genetic variation (13% and 15%) in all populations when controlling for population structure and geography. Across the 27 populations, any single effect of genetic structure and geography only accounts for 9% and 7% of the total neutral genetic variance, respectively (Table S4). Our comparisons of environmental (climate and soil) effects on neutral versus adaptive genetic variation revealed that environmental variables exclusively explained 36% of the adaptive genetic variance, noticeably higher than their 29% contribution to neutral genetic variation.

The GF analysis results showed that population structure and maximum temperature of the warmest month (bio 5) are the most important variables for all populations, while precipitation seasonality (bio 15), precipitation of the warmest quarter (bio 18) and sand are the most important environmental factors related to genetic variance in SC, NK and JA, respectively (Figure S10). The mean diurnal range (bio 2) was also of high importance in both SC and NK. Among the climate variables, allelic composition sharply changes when the mean diurnal range is 7°C–8°C, the precipitation seasonality is 43–60, and the precipitation of the warmest quarter is 600–800 mm (Figure S11). Together, these results suggest that the identified adaptive variants in our study are highly associated with environmental variables, although the confounded effects of population structure and geography were also considered.

### 3.5 Identification and Gene Ontology of GEA SNPs

**FIGURE 4:**
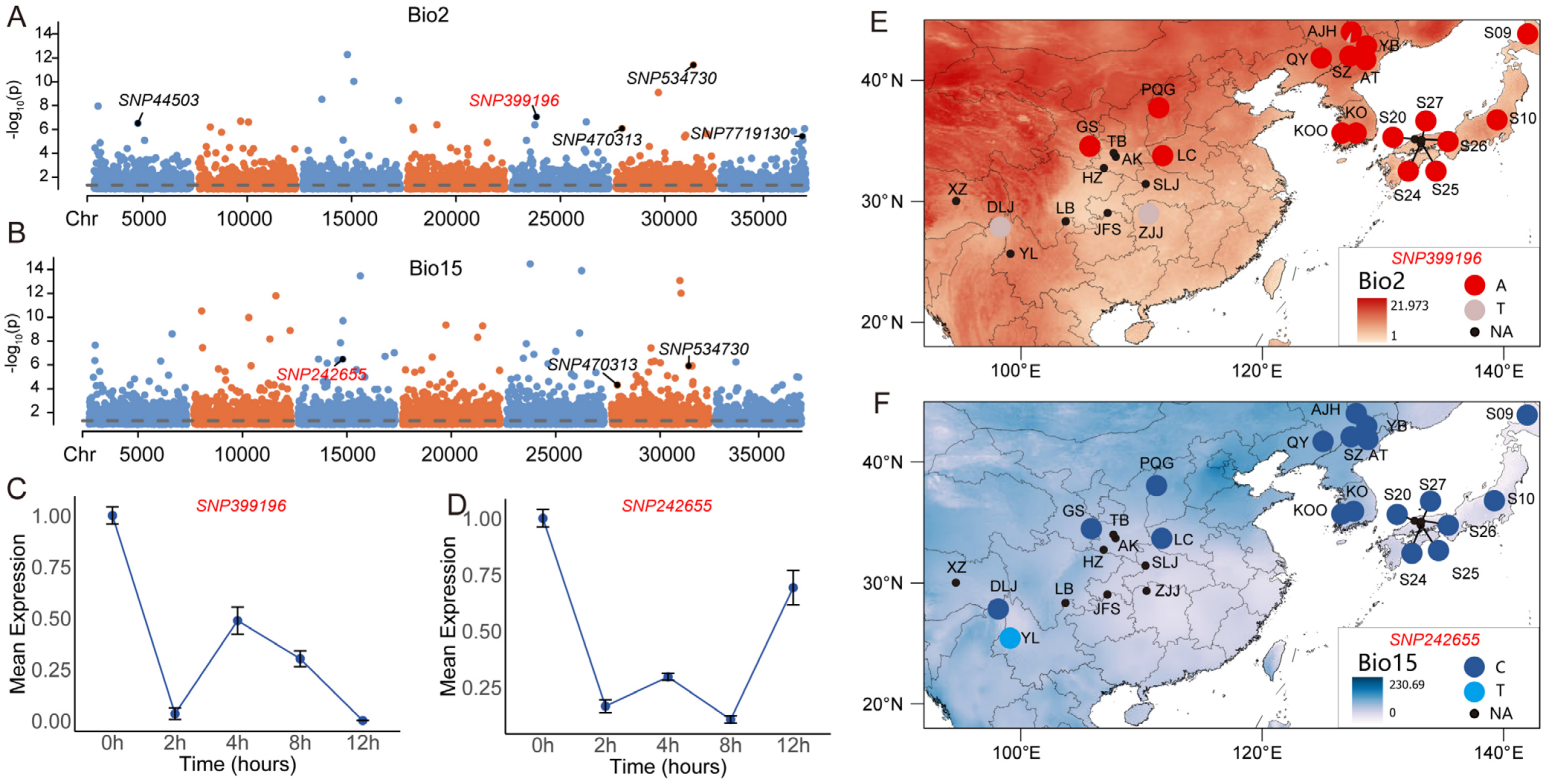
Genome‐wide screening of loci associated with local environmental adaptation, showing a Manhattan plot for variants associated with mean diurnal range of temperature (Bio 2) and precipitation seasonality (Bio 15). The selected candidate SNPs are labelled in their positions, and candidate SNPs were marked in red. The Manhattan plots of candidate adaptive SNPs associated with (A) Bio 2 and (B) Bio 15 are based on LFMM. Dashed horizontal lines represent significance thresholds of FDR = 0.05. Dynamic relative expression level of genes corresponding to (C) SNP 39,9196 and (D) SNP 24,2655 using qRT‐PCR under cold and drought treatments, respectively. Allele variation of candidate adaptive SNPs (SNP 39,9196 and SNP 242655) associated with (E) Bio 2 and (F) Bio 15. Colours on the map are based on variations of the relevant climate variables across the distribution range.

According to the GEA analyses, a total of 262 SNPs and 227 SNPs were associated with one or more environmental variables using the latent factor mixed model (LFMM) and RDA, respectively (Figure S12). Out of the 13 SNPs detected by the two methods, most of them were related to more than one environmental variable (Figure 4; Table S5). To further remove the effects of high genetic structure on GEA SNPs, we ran RDA and LFMM analyses on each of the three groups separately to further screen the candidate loci. A total of 205, 459 and 74 core GEA SNPs were detected in SC, NK and JA, respectively. Alignments of the 13 candidate SNPs across all populations against the complete transcripts of *A. himalaicum* recovered six contigs with an E‐value smaller than 10^−5^. The six transcripts were annotated through BLASTN against the *A. thaliana* reference genome (TAIR v10) to assign putative gene identities (Table S6). Among the successfully annotated genes, SNP 56,633 was located in a homologue of AT1G78950 (beta‐amyrin synthase: *BAS*), SNP 1,93,109 was located in the homologue of AT2G32560 (clock‐regulated, F‐box with a long hypocotyl 1: *CFH1*), SNP 2,11,051 was located in the homologue of AT5G23450 (long‐chain base kinase 1: *LCBK1*), SNP 2,42,655 was located in the homologue of AT3G14570 (glucan synthase‐like 4: *GSL4*), SNP 3,89,447 was located in the homologue of AT5G16980 (Zinc‐binding dehydrogenase family proteins: *ZDs*), and SNP 3,99,196 was located in the homologue of AT5G6000 (heat shock proteins: *HSPs*).

We further explored the signals for geographic distribution of SNPs in the genes under local adaptation. For SNP 3,99,196, the populations carrying the T allele were from SC (DLJ, ZJJ) with a relatively low mean diurnal range, while the other populations were mainly identified with the A allele (Figure 4A,C). In addition to temperature‐related SNPs, we found one precipitation‐related SNP, 2,42,655, from a population of SC (YL) with a relatively low precipitation seasonality carrying the T allele, whereas populations in the northern region were identified with the C allele (Figure 4B,D). Further functional testing following cold stress treatment showed the expression of the gene corresponding to SNP 3,99,196 showed an overall trend of downregulation, despite exhibiting a transient peak at 4 h (Figure 4C). Expression patterns of the gene corresponding to SNP 2,42,655 under drought stress revealed an overall trend of downregulation, although partial recovery of gene expression was observed after 12 h (Figure 4D).

### 3.6 Prediction of Genomic Offset Under Future Climate Change

**FIGURE 5:**
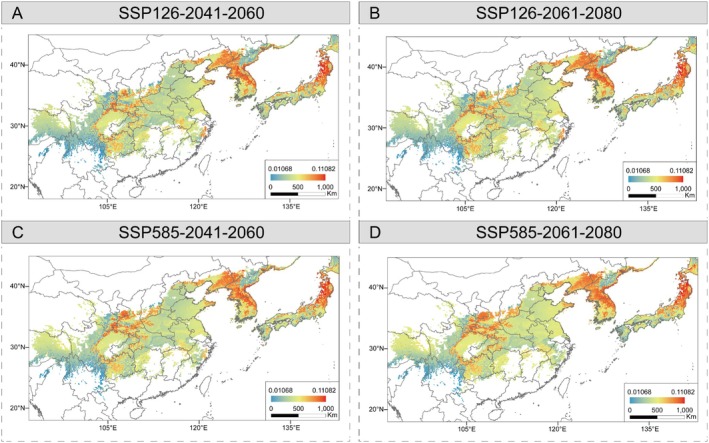
Predicted local genetic offset to future climate change under (A) SSP126 and (C) SSP585 during 2041–2060. Local genetic offsets to future climate changes under (B) SSP126 and (D) SSP585 scenarios during 2061–2080. The colour scale represents low to high levels of genomic offset values.

**FIGURE 6:**
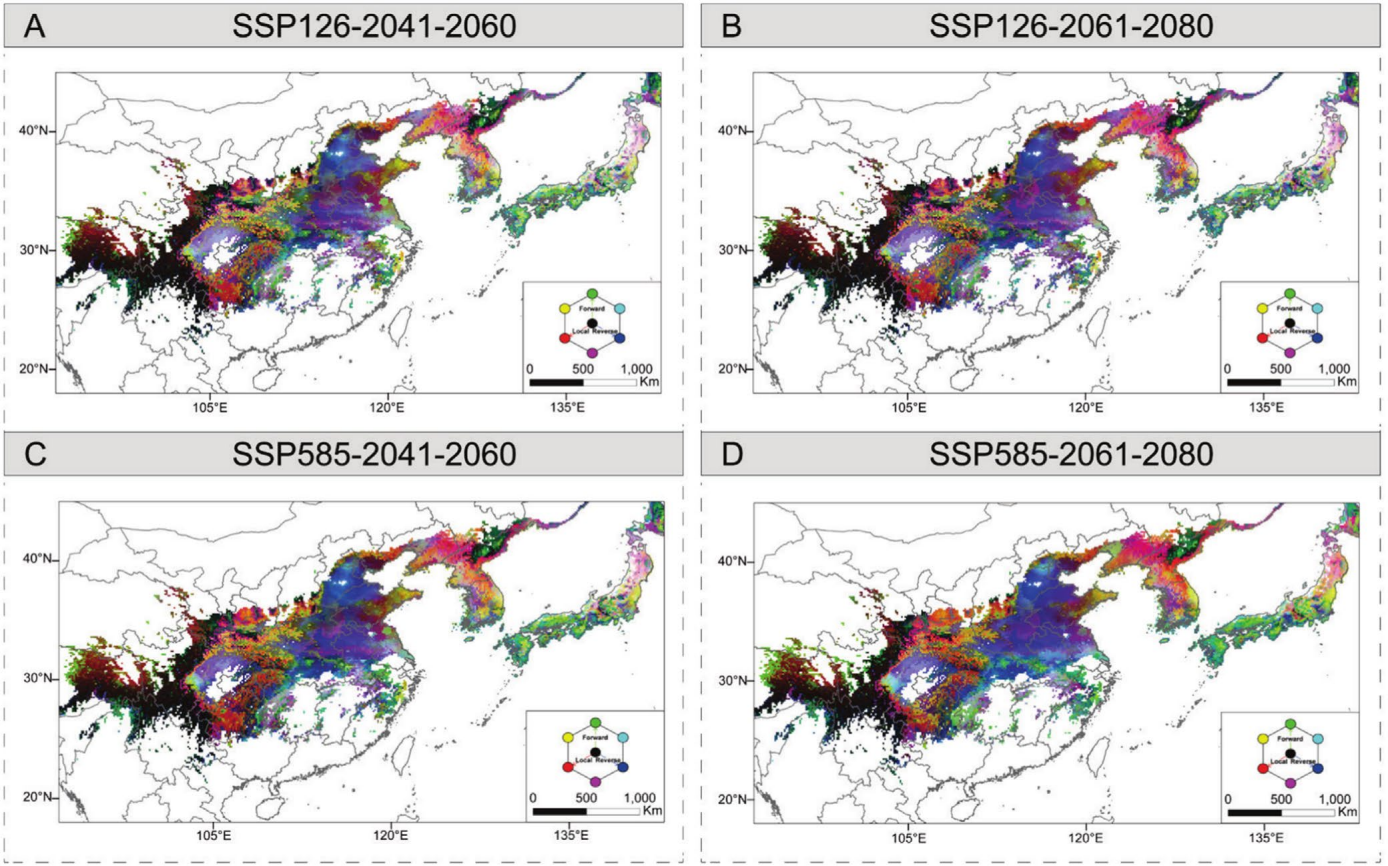
RGB map of local (red), forward (green) and reverse (blue) offsets under (A) SSP126 and (C) SSP585 during 2041–2060. RGB map of local (red), forward (green) and reverse (blue) offsets under (B) SSP126 and (D) SSP585 during 2061–2080. The unlimited dispersal distance was used for the forward offsets.

**FIGURE 7:**
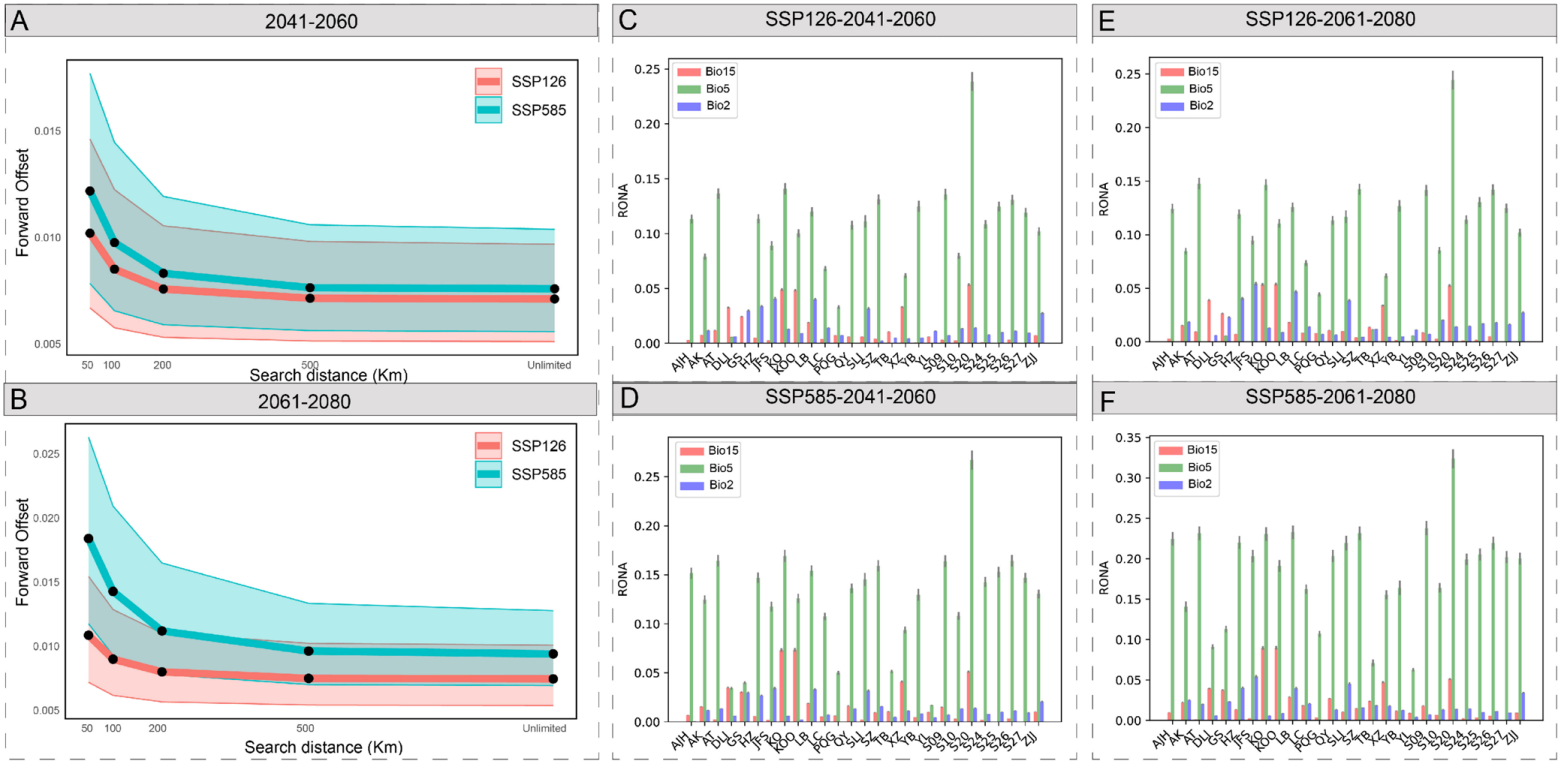
Comparison of the genetic offsets and risk of nonadaptedness plot (RONA) to future climate changes under two different climate scenarios (SSP126 and SSP585). The forward genetic offsets under two different climate scenarios (SSP126 and SSP585) under (A) 2041–2060 and (B) 2061–2080 when the maximum allowable migration was limited to 50, 100, 200, 500 km and unlimited distance. Risk of nonadaptedness plot (RONA) for three important environmental factors under SSP126 and SSP585 in (C, D) 2041–2060 and (E, F) 2061–2080, with bars and lines representing weighted means and standard error for each population, respectively.

Based on the turnover in allele frequencies between present and future environmental gradients, our results found a highly similar spatial pattern of genomic offset between 2041–2160 and 2061–2080 based on all neutral SNPs and 13 core adaptive SNPs (Figures 5, 6 and S13–S18). Among the 27 populations, most present a higher genomic vulnerability with increasing emission (Figure 7A,B). In general, low local offsets are predicted in the eastern Himalaya populations and most of the central populations, suggesting that populations experience minimal future disruption to the genotype‐climate association at these locations. In contrast, the southeastern edge of the Himalayas, near the Sichuan Basin, southern Northeast China and the northern Korean Peninsula, as well as northern Japan, are considered the most vulnerable regions with relatively high local offsets (Figure 5). To integrate the effects of migration to quantify the maladaptation of populations, we further assessed forward genetic offset under different dispersal distances (50, 100, 200, 500 km and unlimited) by identifying the minimum predicted offset within the migration range. The forward offset decreases by ca. 14.52% and ca. 10.44% when the maximum migration distance was set from 50 to 100 km and from 100 to 200 km, respectively (Figure 7A,B). Furthermore, high reverse offsets in the Sichuan Basin, the northern Korean Peninsula and the areas bordering China, and northern Japan suggest that few contemporary populations are pre‐adapted to the projected future climates in these regions (Figures 6 and S14). The results reveal high correlations between the genetic offsets and climatic variables of precipitation seasonality, precipitation of the warmest quarter and the mean diurnal range, which is consistent with R^2^ weight importance and the cumulative importance of allelic change by the GF analysis (Figure S19). We also calculated the risk of non‐adaptedness (RONA) to predict the maladaptation of populations under future climate conditions, and the average RONA levels are consistent across different climate models (Figure 7C–F). The maximum temperature of the warmest month (bio 5) is the most represented variable by RONA, and population S20 from JA has the lowest adaptedness potential for bio 5 compared to other populations under future climates. In addition, the RONA results are largely consistent with genetic offsets, such as populations YB, KO, KOO, JFS and XZ with higher RONA values located in regions of high genetic offsets.

## 4 Discussion

### 4.1 Heterogeneous Landscape Effects on the Genomic Variation in *A. himalaicum*

Understory herbs are playing a critical role in maintaining biodiversity and ecological integrity in East Asian forests, while their distribution patterns reflect the sustainability of forest management practices and serve as key indicators for conserving herbaceous plant communities in this region (Deng et al. 2023). However, the relative roles of demographic history and ecological adaptation to a heterogeneous landscape in shaping genetic variation and evolutionary risk for understory herbs remain unknown. The late Miocene–Pliocene climate changes were found to be driven by the uplift of the Qinghai‐Xizang Tibetan Plateau, likely triggering the early population divergence of *A. himalaicum* in East Asia (Deng et al. 2018). Our current results show that the ancient divergence of the three groups was estimated at c. 4.08 Mya (Figure 2; Table S3), which is consistent with the monsoon intensification and the formation of the arid belt between 35° N and 45° N (Bai et al. 2016; Guo et al. 2008). The split of the NK and JA groups approximately 0.48 Mya is closely associated with the disappearance of the East China Sea Land Bridge during the Pleistocene (Qiu et al. 2009; Sakaguchi et al. 2012). One caveat is that our genetic divergence estimates contain uncertainties due to the large variation of the fossil calibration (Deng et al. 2018). Consequently, these temporal estimates should be interpreted with caution in an evolutionary application. According to inferences of effective population changes (Figure 2; Table S3), the SC group has experienced an expansion–shrinkage process from 0.39 Mya to 0.32 Mya, likely due to climate oscillations during the Pleistocene (Qiu et al. 2009). In contrast, the populations from the NK group underwent a recent shrinkage–expansion from 90.6 kya to 5.25 kya, which may be related to climate fluctuations and human activities. The JA group experienced a significant decrease, corresponding to lower genetic diversity, likely due to founder events along with the route of divergence and colonisation.

Notably, few studies have explored the effects of local adaptation to ecological factors after historical phylogeographic divergence of herbaceous plants in East Asia (e.g., the formation of arid zones and the East China Sea Land Bridge; Bai et al. 2016; Sakaguchi et al. 2012). Previous studies have revealed that gene flows exist between the northern and southern populations in the arid zone, and a hybrid zone is expected in the north–south arid belt. However, the genome‐wide SNPs of this study recover a strong spatial genetic structure with well‐delimited genetic groups, without distinct mixture events across *A. himalaicum* populations (Figure 1). Therefore, it can be inferred that long‐term local adaptation to heterogeneous precipitation and temperature could have contributed to observed genetic differentiation between genetic lineages (Thuiller 2004). During the Miocene to Pliocene, the intensification of monsoonal systems and increased aridity led to a pronounced climatic divergence between southern and northern East Asia (Guo et al. 2008). This persistent environmental differentiation drove the evolution of distinct regional adaptations, with southern populations developing specialised traits for hot‐humid monsoonal conditions, while their northern counterparts adapted to cold‐dry climatic regimes (Bai et al. 2016; Cao et al. 2020; Donoghue et al. 2001). A significant IBE result in this study infers the genetic divergences have been driven by climatic factors, which is shown as a lower correlation than the IBD result (Figure 3). Although genetic structure and spatial vectors participated in shaping the spatial distribution of genomic variation across populations (Table S4), our corrected RDA further verifies that genetic variations are largely explained by climatic variables across all populations and within each genetic group. In addition, a stronger IBE and higher amount of genetic variance, which can be explained by climatic factors in adaptive variance, also support population differences driven by climate adaptation (Table S4). These include extreme temperature and precipitation variables, such as the mean diurnal range (bio 2), maximum temperature of the warmest month (bio 5) and precipitation of the warmest quarter (bio 18; Table S2). The mean diurnal range has an important effect on plant stress resistance and phenological development (Gallou et al. 2023; Jackson and Forster 2010). In *A. himalaicum*, significant divergence in mean diurnal range is observed between northern and southern populations (11.36 vs. 8.76, *p* < 0.05), suggesting potential local adaptation. Since photosynthesis and respiration rates vary across growth periods, shifts in mean diurnal range may disrupt the metabolic balance, ultimately affecting plant growth and fitness (Chen et al. 2017; Xiao et al. 2017). This adaptation to diurnal thermal fluctuations could explain the ability to thrive across a broad distribution, particularly at the northern edge of its East Asian range. In addition to temperature, extreme precipitation changes (i.e., precipitation of the warmest quarter) were also found to be closely associated with genomic variation in *A. himalaicum* populations (Figure 3 and Table S4). The Asian monsoon climate drives seasonal precipitation differences (Chen et al. 2013), with southern populations of *A. himalaicum* experiencing higher warmest‐quarter precipitation than northern populations in China (473.08 vs. 441.67; *p* < 0.05). Such extreme precipitation regimes act as one of the key abiotic stressors, likely selecting for enhanced water use efficiency. Similar adaptive mechanisms have been documented in other species, including *Quercus aquifolioides* and *Cephalotaxus oliveri* (Du et al. 2020; Siepielski et al. 2017; Wang et al. 2016), suggesting a broader evolutionary pattern under monsoon‐driven climatic variability. Taken together, these findings highlight the potential importance of extreme precipitation and temperature as selective pressures in shaping the genetic structure of *A. himalaicum*.

### 4.2 Genomic Outliers Associated With Local Climate Adaptation and Transient Response from qRT‐PCR

With rapid global changes, the climate's impact on biodiversity is primarily determined by the adaptive capacity of populations (Brown et al. 2016; Usinowicz and Levine 2021). We identified 13 candidate loci by two GEA analyses, which represent 2.7% of all outliers detected by at least one of the analyses across all populations (Figure S12). While the integration of structure‐corrected LFMM and RDA yielded 13 candidate SNPs associated with environmental adaptation, it is important to acknowledge that this conservative approach inherently prioritises minimising false positives at the potential cost of statistical power. The requirement for concordance between LFMM and RDA results for effectively reducing spurious associations can overlook many GEA candidate SNPs detected by only one method due to differences in their underlying assumptions. The incorporation of population structure corrections and multivariate constraints in RDA increases model complexity, which may disproportionately reduce sensitivity to loci with weaker selection signals (Capblancq and Forester 2021). Furthermore, stringent multiple‐testing corrections in LFMM (i.e., FDR) applied to genome‐wide datasets likely excluded loci with moderate adaptive effects (Caye et al. 2019; Frichot and François 2015). Given the limitation of genomic resources for *A. himalaicum* and the RAD‐seq approach used in the current study, only a very small portion of potential selective loci of *A. himalaicum* were likely identified. Nonetheless, several of the SNPs were found to be associated with temperature and precipitation variables, as well as showing geographic distributions in allelic variation (Figure 4). For example, SNP 3,99,196, located in a homologue of heat shock protein (*HSP*), is closely associated with mean diurnal range (bio2), suggesting a role in adaptation to extreme temperature. The superfamily of *HSPs* plays crucial roles in cellular homeostasis by preserving membrane integrity, regulating protein conformation and mediating organellar protein transport (Kazemi‐Shahandashti and Maali‐Amiri 2018). Our analysis revealed that SNP 3,99,196, which occurs in populations DLJ and ZJJ in areas with lower diurnal temperature ranges, possesses a unique alternate allele. qRT‐PCR experimental results suggest this gene may function in short‐term response to cold stress (Figure 4). Although *HSP* expression was not induced under cold stress in our experiments, we propose that this gene may play a crucial role in regulating extreme temperature tolerance in marginal habitats. Meanwhile, we have identified one precipitation‐associated locus, SNP 2,42,655, annotated as an ortholog to *GSL4* (Figure 4), associated with the precipitation seasonality (bio 15). The glucan synthase‐like (*GSLs*) gene family encodes critical enzymes to catalyse the biosynthesis of callose (β‐1,3‐glucan) from UDP‐glucose substrates, which regulates callose deposition in response to different developmental, physiological and environmental signals in various plant tissues (Zhu et al. 2010). Our qRT‐PCR results further reveal its potential functions in combating drought stress, suggesting the crucial physiological functions in plant development. However, it is critical to emphasise that transient transcriptional changes under short‐duration treatments exhibit inherent instability, which may introduce stochastic noise. Notably, the SNPs identified through RAD‐seq may not represent true causal variants but loci that have genetic linkage (LD) with undetected functional variations of indels in promoters or SNPs with changes in chromatin openness in enhancer regions. Thus, genomic‐level validation is essential to assess their potential links to circadian rhythms or stress responses. For cost‐effectiveness and to ensure independence from a reference genome, the widely used RAD‐seq approach was applied in the current study. RAD‐seq has been widely used in studies of ecological, evolutionary and conservation genomics for non‐model species, but this approach presents inherent limitations for studying adaptive evolution (Andrews et al. 2016). Under some conditions, RAD‐seq may incur restriction site‐associated bias and a lack of chromosomal‐level genome may obscure the selection of key adaptive loci, particularly in regulatory regions and structural variants not linked to enzyme recognition sites. Furthermore, the limited marker density reduces the ability to distinguish selection signals from background linkage disequilibrium, potentially inflating false‐positive associations between candidate loci and climatic variables. Future studies that integrate long‐read sequencing, epigenomics and functional characterisation through CRISPR‐Cas9 editing or knockout models could clarify the regulating roles of these genes in local adaptation. Despite these caveats, the identified candidate loci associated with environmental adaptation in *A. himalaicum* can be used as genetic resources for conservation breeding for understory herbs.

### 4.3 Prediction of Genomic Vulnerability and Conservation Implications

In comparison with traditional niche modelling methods, genetic vulnerability can predict spatial survival regions by using genotype–environment relationships under future climate scenarios (Ellis et al. 2012; Siepielski et al. 2017). Based on the GF and RONA results under different climate models, we have estimated the multiple metrics of maladaptation, combining migration distances to sketch a spatial distribution pattern for the most preadapted or maladapted under future climate change (Figure 5). As expected, genetic offsets increased across populations under more severe climate change scenarios (Figure 5; 2041–2060 vs. 2061–2080) and higher emissions (Figure 5; SSP585 vs. SSP126), inferring ongoing threats and the urgency of protection. The spatiotemporal genomic vulnerability is closely related to genetic diversity among the three clusters (Figures 5, 6 and S7), inferring that the patterns of genetic diversity within *A. himalaicum* are associated with the landscape heterogeneity under climate changes. We have found that marginal populations of *A. himalaicum*, such as those in the southeastern Himalayas, the northern Korean Peninsula and northern Japan, exhibit a high level of genetic vulnerability, suggesting that these populations are potentially at high risk of extinction under future climate changes. The high genetic vulnerability of populations at the longitudinal/latitudinal margins across the distribution range partly reflects the fact that the effects of extreme climatic variables (e.g., mean diurnal range and precipitation of the warmest quarter) are the main drivers of genetic variance of *A. himalaicum*. These edge populations, chronically exposed to climatic extremes with a relatively low genetic diversity, may accumulate genetic loads and result in reduced adaptive plasticity under climatic changes. The combined prediction of local, forward and reverse offsets varies throughout the range of *A. himalaicum*, but those populations in the southeastern Himalayas, the northern Korean Peninsula, and northern Japan are less resilient to future climate. This suggests that no populations in this region, either locally or elsewhere within the range of this species, are preadapted to future climates. Meanwhile, populations in these regions are predicted to face the highest risk of extinction, as their genetic vulnerability cannot be mitigated by dispersal to other suitable habitats (Figure 6). Importantly, certain southern populations (e.g., XZ and YL) harbour unique climate‐adaptive alleles associated with extreme climate resilience, underscoring the critical conservation priority of these populations. Our predictions of population vulnerability to climate changes provide more precise direction in conservation and restoration efforts. Further artificial facilitation of gene flow between populations and those harbouring complementary adaptive haplotypes for cross‐regional transplantation may be helpful to enhance population adaptation (Gougherty et al. 2021). By quantifying the maximum dispersal capacity of *A. himalaicum*, it is possible to assist gene flow and migration for central Chinese populations by translocating individuals to climatically suitable habitats within and beyond the current ranges. However, it is worth emphasising that designing experimental validation remains crucial for refining climate‐response predictions and optimising conservation interventions. Although genetic offset provides a useful proxy for predicting population maladaptation, it primarily measures allele frequency shifts under projected climate change rather than assessing direct fitness consequences. These allele frequency shifts may overestimate or underestimate actual extinction risk if critical adaptive loci are inadequately represented in the analysis. Current genetic offset estimates should be interpreted with caution, particularly when small frequency shifts coincide with high environmental change. Future studies should incorporate fitness‐related traits to validate genetic offset predictions. Most importantly, rigorous validation requires reciprocal transplant experiments employing multiple genotypes across diverse environments (Lotterhos 2024). In the future, conservation strategies would benefit from combining genomic predictions with additional predictive indicators and transplanting experiments, thereby developing more robust management approaches.

## Author Contributions

T.D. and H.W. conceived and designed this study. N.L., X.H., Q.L. and X.W. collected the materials. N.L. and Y.W. analysed the data. Y.H. and Y.W. helped with the experiments. N.L. wrote the manuscript; X.H. and T.D. reviewed the manuscript. All authors read and approved the final manuscript.

## Ethics Statement

All international, national and institutional guidelines for the care and use of plants were followed.

## Conflicts of Interest

The authors declare no conflicts of interest.

## Supporting information


**Data S1:** mec70068‐sup‐0001‐Supinfo01.docx.

## Data Availability

The raw sequencing data related to the current study have been deposited in the NCBI GenBank (BioProject: PRJNA662661). The SNPs dataset used for analyses has been deposited in Dryad (https://doi.org/10.5061/dryad.j6q573nq6). The code used in this study is available in GitHub (https://github.com/LN‐11/LN_Ade/tree/main/code).
