# Supplementary material for: Genomic Insights Into Local Adaptation Across Heterogeneous Understory Habitats and Climate Change Vulnerability
Source: Mol Ecol. 2025 Aug 18;34(19):e70068. doi: 10.1111/mec.70068 (PMC12456111; doi:10.1111/mec.70068)

**Supplemental Information for:**

**Genomic insights into local adaptation across heterogenous**

**understory habitats and climate change vulnerability**

Nan Lin^1,2,3^ Yakun Wang^2^, Xiankun Wang^4^, Yuxuan He^2^, Xianhan Huang^1^, Qun Liu^1^, Hengchang Wang^5,*^, Tao Deng^1,*^

^1^State Key Laboratory of Plant Diversity and Specialty Crops, Kunming Institute of Botany, Chinese Academy of Sciences, Kunming 650201, Yunnan, China

^2^College of Life Science, Henan Agricultural University, Zhengzhou 450046, Henan, China

^3^Henan Engineering Research Center for Osmanthus Germplasm Innovation and Resource Utilization, Henan Agricultural University, Zhengzhou 450046, Henan, China

^4^College of Landscape Architecture and Art, Henan Agricultural University, Zhengzhou 450046, Henan, China

^5^CAS Key Laboratory of Plant Germplasm Enhancement and Specialty Agriculture, Wuhan Botanical Garden, Chinese Academy of Sciences, Wuhan 430074, Hubei, China.

*Authors for correspondence:

Hengchang Wang: hcwang@wbgcas.cn

Tao Deng: dengtao@mail.kib.ac.cn

**Table of Contents:**

| **Table S1** | **Page 3** |
| --- | --- |
| **Table S2** | **Page 5** |
| **Table S3** | **Page 7** |
| **Table S4** | **Page 8** |
| **Table S5** | **Page 13** |
| **Table S6** | **Page 14** |
| **Fig. S1** | **Page 16** |
| **Fig. S2** | **Page 16** |
| **Fig. S3** | **Page 17** |
| **Fig. S4** | **Page 17** |
| **Fig. S5** | **Page 18** |
| **Fig. S6** | **Page 18** |
| **Fig. S7** | **Page 19** |
| **Fig. S8** | **Page 19** |
| **Fig. S9** | **Page 20** |
| **Fig. S10** | **Page 21** |
| **Fig. S11** | **Page 22** |
| **Fig. S12** | **Page 22** |
| **Fig. S13** | **Page 23** |
| **Fig. S14** | **Page 24** |
| **Fig. S15** | **Page 24** |
| **Fig. S16** | **Page 25** |
| **Fig. S17** | **Page 26** |
| **Fig. S18** | **Page 26** |
| **Fig. S19** | **Page 27** |

Table S1 Detailed information of populations, number of individuals in each population, population locations, and statistics of genetic variability for 27 populations of *Adenocaulon* *himalaicum*; Obs_Het, observed heterozygosity; Exp_Het, expected heterozygosity, Pi, nucleotide diversity; *F*_IS_, inbreeding coefficient.

| **Populations** | **Number of individuals** | **Latitude (^。^N)** | **Longitude (^。^E)** | **Altitude (m)** | **Obs_Het** | **Exp_Het** | **Pi** | ***F*_IS_** |
| --- | --- | --- | --- | --- | --- | --- | --- | --- |
| AJH | 8 | 44.04 | 127.76 | 489 | 0.0262 | 0.0735 | 0.0786 | 0.1334 |
| AK | 7 | 33.69 | 107.86 | 1965 | 0.0335 | 0.0304 | 0.0329 | 0.016 |
| AT | 8 | 42.4 | 128.1 | 773 | 0.0272 | 0.047 | 0.0503 | 0.0589 |
| DLJ | 4 | 27.7 | 98.35 | 1474 | 0.0185 | 0.0104 | 0.0119 | -0.0116 |
| GS | 4 | 34.37 | 106.01 | 1506 | 0.0164 | 0.0097 | 0.0111 | -0.0094 |
| HZ | 9 | 32.75 | 106.87 | 1451 | 0.0348 | 0.0477 | 0.0507 | 0.0302 |
| JFS | 5 | 29.04 | 107.18 | 1824 | 0.0296 | 0.018 | 0.02 | -0.0176 |
| KO | 9 | 35.82 | 127.33 | 578 | 0.0196 | 0.0628 | 0.0668 | 0.1133 |
| KOO | 5 | 35.9 | 127.34 | 497 | 0.0179 | 0.0586 | 0.0651 | 0.1027 |
| LB | 10 | 28.34 | 103.71 | 1850 | 0.0282 | 0.0404 | 0.0426 | 0.0361 |
| LC | 10 | 33.73 | 111.66 | 1522 | 0.017 | 0.0103 | 0.0108 | -0.0117 |
| PQG | 8 | 37.85 | 111.46 | 1844 | 0.0323 | 0.0305 | 0.0326 | 0.0054 |
| QY | 10 | 41.85 | 124.94 | 580 | 0.0209 | 0.0615 | 0.0653 | 0.1174 |
| SLJ | 4 | 31.43 | 110.34 | 213 | 0.0269 | 0.0155 | 0.0177 | -0.016 |
| SZ | 6 | 42.41 | 127.66 | 634 | 0.022 | 0.0532 | 0.058 | 0.0871 |
| TB | 7 | 34 | 107.68 | 1030 | 0.0314 | 0.0353 | 0.0381 | 0.0231 |
| XZ | 6 | 30.03 | 94.63 | 550 | 0.0221 | 0.0131 | 0.0143 | -0.0145 |
| YB | 8 | 42.48 | 128.67 | 606 | 0.0239 | 0.046 | 0.0493 | 0.0738 |
| YL | 2 | 25.66 | 99.13 | 499 | 0.023 | 0.0134 | 0.0178 | -0.0077 |
| S09 | 15 | 43.79 | 142.3 | 705 | 0.0156 | 0.0098 | 0.0102 | -0.0105 |
| S10 | 15 | 36.75 | 139.59 | 2235 | 0.0176 | 0.0352 | 0.0365 | 0.0759 |
| S20 | 17 | 35.14 | 132.62 | 865 | 0.0258 | 0.0636 | 0.0657 | 0.1109 |
| S24 | 11 | 34.79 | 133.14 | 2619 | 0.0192 | 0.0121 | 0.0127 | -0.0125 |
| S25 | 6 | 34.82 | 133.13 | 3024 | 0.0216 | 0.0252 | 0.0275 | 0.021 |
| S26 | 7 | 34.83 | 133.12 | 1469 | 0.0203 | 0.0135 | 0.0147 | -0.0106 |
| S27 | 10 | 35.07 | 133.18 | 2110 | 0.019 | 0.0408 | 0.0431 | 0.0896 |
| ZJJ | 10 | 29.33 | 110.43 | 1001 | 0.0224 | 0.0142 | 0.015 | -0.0142 |

Table S2 The 19 bioclimatic variables under current conditions (1970-2000) and five soil factors were extracted from WorldClim v2.1 and the Harmonized World Soil Database v1.21 raster layers at 30 s (~1 km^2^) resolution.

| **Environmental variables** | **Description of environmental variables** |
| --- | --- |
| Bioclimatic variables | |
| bio 1 | Annual mean temperature (℃) |
| bio 2 | Mean diurnal temperature range (℃) |
| bio 3 | Isothermality (BIO2/BIO7×100) |
| bio 4 | Temperature seasonality (standard deviation ×100) |
| bio 5 | Maximum temperature of warmest month (℃) |
| bio 6 | Minimum temperature of coldest month (℃) |
| bio 7 | Temperature annual range (℃) |
| bio 8 | Mean temperature of wettest quarter (℃) |
| bio 9 | Mean temperature of driest quarter (℃) |
| bio 10 | Mean temperature of warmest quarter (℃) |
| bio 11 | Mean temperature of coldest quarter (℃) |
| bio 12 | Annual precipitation (mm) |
| bio 13 | Precipitation of wettest month (mm) |
| bio 14 | Precipitation of driest month (mm) |
| bio 15 | Precipitation seasonality (coefficient of variation) |
| bio 16 | Precipitation of wettest quarter (mm) |
| bio 17 | Precipitation of driest quarter (mm) |
| bio 18 | Precipitation of warmest quarter (mm) |
| bio 19 | Precipitation of coldest quarter (mm) |
| Soil variables | |
| CEC | Cation exchange capacity of soil |
| OCD | Carbon density of soil |
| sand | The content of sand |
| TAXNWRB | The taxonomic class in the World Reference Base system |
| PH_H2O | The pH of soil |

Table S3 Parameter estimates with 95% highest posterior density (HPD) intervals for the best model of *Adenocaulon* *himalaicum.*

| **Parameter** | **Point Estimation** | **Median Estimation** | **95% CI lower bound** | **95% CI upper bound** |
| --- | --- | --- | --- | --- |
| N1 | 64,200,227 | 38,894,999 | 35,771,594 | 42,807,877 |
| N2 | 4,945,367 | 812,759 | 139,805 | 4,143,512 |
| NPOPNK | 14,811,092 | 964,444 | 422,984 | 260,486,964 |
| NNEXP | 514,984 | 427,449 | 56,415 | 771,979 |
| ACN | 77,872,109 | 35,181,427 | 4,616, 755 | 197,583,242 |
| NPOPJ0 | 317,273 | 258,661 | 38,245 | 449,156 |
| NJKEXP | 25,460,464 | 3,567,995 | 237,813 | 82,126,937 |
| ACJ | 262,915,378 | 85,119,872 | 1,439,568 | 7,959,469,604 |
| NPOPSC | 678,494 | 611,095 | 206,826 | 122,332, 071 |
| NSCEXP | 148,471,682 | 29,952,667 | 1,003,380 | 542,345,075 |
| ACS | 3,617,996 | 736,759 | 212,568 | 3,952,482 |
| MIGRNJ | 3.12E-07 | 4.36E-07 | 2.36E-07 | 8.22E-07 |
| MIGRJN | 5.97E-07 | 7.90E-07 | 4.01E-07 | 8.62E-07 |
| MIGA2S | 8.15E-10 | 4.54E-08 | 3.77E-10 | 5.34E-07 |
| MIGSA2 | 4.90E-10 | 5.38E-08 | 3.04E-10 | 5.17E-07 |
| MIGASN | 1.08E-08 | 7.26E-09 | 5.05E-10 | 8.55E-08 |
| MIGANS | 7.14E-07 | 5.91E-07 | 3.17E-07 | 8.63E-07 |
| T1 | 4,085,488 | 1,188,003 | 570,357 | 3,900,256 |
| T2 | 487,127 | 524,644 | 209,951 | 1, 270,841 |
| T3 | 46,781 | 34,005 | 8,260 | 67,645 |
| TNKBOT | 5,256 | 51,934 | 239 | 154,025 |
| TNKEND | 90,609 | 101,328 | 40,014 | 194,307 |
| TJAKBOT | 400,825 | 274,315 | 269 | 609,704 |
| TJAKEND | 451,247 | 433,303 | 152,820 | 979,202 |
| TSCBOT | 324,060 | 214,233 | 476 | 887,028 |
| TSCEND | 394,434 | 309,461 | 11,93 | 1,159,305 |

Table S4 Results of partitioning of the variance associated with climate variables, soil variables, geography and neutral genetic structure based on partial redundancy analysis (pRDA). The proportion of explainable variance represents the total constrained variation explained by the full model. “^***^” refers to p ≤ 0.001 and NS refers to not significant.

| **Partial RDA model** | **Inertia** | **R^2^ (adj)** | **Pr (>F)** | **Proportion of**  **explained variance** | **The proportion of**  **total variance** |
| --- | --- | --- | --- | --- | --- |
| **All populations** | | | | | |
| ***Adaptive SNPs*** | | | | | |
| Full F~clim. + struct. + soil + geog. | 11.41 | 0.87 | 0.001*** | 1.00 | 0.8777 |
| Full F~clim. \| (struct. + soil + geog.) | 1.60 | 0.13 | 0.001*** | 0.15 | 0.1227 |
| Full F~struct. \| (clim. + soil + geog.) | 1.15 | 0.09 | 0.001*** | 0.11 | 0.0884 |
| Full F~soil \| (struct. + clim. + geog.) | 3.07 | 0.25 | 0.001*** | 0.28 | 0.2365 |
| Full F~geog. \| (struct. + clim. + soil) | 0.55 | 0.04 | 0.001*** | 0.05 | 0.0424 |
| Full F~clim. + soil \| (struct. + geog.) | 4.67 | 0.36 | 0.001*** | 0.41 | 0.3595 |
| Confounded clim. + struct. + soil + geog. | 5.04 |  |  | 0.20 | 0.3876 |
| Total unconstrained | 1.62 |  |  |  | 0.1246 |
| Total inertia | 13.00 |  |  |  | 1.00 |
| ***Neutral SNPs*** | | | | | |
| Full F~clim. + struct. + soil + geog. | 19403.45 | 0.53 | 0.001*** | 1.00 | 0.56 |
| Full F~clim. \| (struct. + soil + geog.) | 4544.11 | 0.13 | 0.001*** | 0.24 | 0.13 |
| Full F~struct. \| (clim. + soil + geog.) | 2957.28 | 0.08 | 0.001*** | 0.16 | 0.09 |
| Full F~soil \| (struct. + clim. + geog.) | 5007.79 | 0.14 | 0.001*** | 0.27 | 0.15 |
| Full F~geog. \| (struct. + clim. + soil) | 2507.24 | 0.07 | 0.001*** | 0.13 | 0.07 |
| Full F~clim. + soil \| (struct. + geog.) | 10093.23 | 0.28 | 0.001*** | 0.53 | 0.29 |
| Confounded clim. + struct. + soil + geog. | 4565.19 |  |  | 0.20 | 0.13 |
| Total unconstrained | 14618.25 |  |  |  | 0.42 |
| Total inertia | 34398.00 |  |  |  | 1.00 |
| **Group SC** | | | | | |
| ***Adaptive SNPs*** | | | | | |
| Full F~clim. + soil + geog. | 200.47 | 0.9969 | 0.001*** | 1.000 | 0.9973 |
| Full F~clim. \| (struct. + geog.) | 20.58 | 0.1172 | 0.001*** | 0.118 | 0.0057 |
| Full F~soil \| (clim. + geog.) | 11.72 | 0.0668 | 0.001*** | 0.067 | 0.0583 |
| Full F~geog. \| (clim. + soil) | 0.00 | 0.0000 | - | 0.000 | 0.0000 |
| Confounded clim. + soil + geog. | 168.16 |  |  | 0.815 | 0.8366 |
| Total unconstrained | 1.92 |  |  |  | 0.0095 |
| Total inertia | 201.00 |  |  |  | 1.0000 |
| ***Neutral SNPs*** | | | | | |
| Full F~clim. + soil + geog. | 27760.00 | 0.7874 | 0.001*** | 1.000 | 0.8177 |
| Full F~clim. \| (struct. + geog.) | 4343.88 | 0.1427 | 0.001*** | 0.181 | 0.1280 |
| Full F~soil \| (clim. + geog.) | 1189.42 | 0.0363 | 0.001*** | 0.046 | 0.0350 |
| Full F~geog. \| (clim. + soil) | 0.00 | 0.0000 | - | 0.000 | 0.0000 |
| Confounded clim. + soil + geog. | 22226.70 |  |  | 0.773 | 0.6547 |
| Total unconstrained | 6186.00 |  |  |  | 0.1822 |
| Total inertia | 33947.00 |  |  |  | 1.0000 |
| **Group NK** | | | | | |
| ***Adaptive SNPs*** | | | | | |
| Full F~clim. + soil + geog. | 441.08 | 0.9580 | 0.001*** | 1.000 | 0.9631 |
| Full F~clim. \| (struct. + geog.) | 49.72 | 0.1209 | 0.001*** | 0.126 | 0.0000 |
| Full F~soil \| (clim. + geog.) | 54.80 | 0.17 | 0.001*** | 0.174 | 0.1197 |
| Full F~geog. \| (clim. + soil) | 0.00 | 0.0000 | - | 0.000 | 0.0000 |
| Confounded clim. + soil + geog. | 336.56 |  |  | 0.700 | 0.7348 |
| Total unconstrained | 6.29 |  |  |  | 0.0137 |
| Total inertia | 458.00 |  |  |  | 1.0000 |
| ***Neutral SNPs*** | | | | | |
| Full F~clim. + soil + geog. | 23832.39 | 0.6652 | 0.001*** | 1.000 | 0.7054 |
| Full F~clim. \| (struct. + geog.) | 3381.63 | 0.1070 | 0.001*** | 0.161 | 0.1001 |
| Full F~soil \| (clim. + geog.) | 3735.17 | 0.12 | 0.001*** | 0.179 | 0.1106 |
| Full F~geog. \| (clim. + soil) | 0.00 | 0.0000 | - | 0.000 | 0.0000 |
| Confounded clim. + soil + geog. | 16715.59 |  |  | 0.661 | 0.4947 |
| Total unconstrained | 9955.00 |  |  |  | 0.2946 |
| Total inertia | 33787.00 |  |  |  | 1.0000 |
| **Group JA** | | | | | |
| ***Adaptive SNPs*** | | | | | |
| Full F~clim. + soil + geog. | 70.95 | 0.9696 | 0.001*** | 1.000 | 0.9719 |
| Full F~clim. \| (struct. + geog.) | 0.00 | 0.0000 | - | 0.000 | 0.0000 |
| Full F~soil \| (clim. + geog.) | 0.00 | 0.0000 | - | 0.000 | 0.0000 |
| Full F~geog. \| (clim. + soil) | 0.00 | 0.0000 | - | 0.000 | 0.0000 |
| Confounded clim. + soil + geog. | 70.95 |  |  | 1.000 | 0.9719 |
| Total unconstrained | 2.05 |  |  |  | 0.0281 |
| Total inertia | 73.00 |  |  |  | 1.0000 |
| ***Neutral SNPs*** | | | | | |
| Full F~clim. + soil + geog. | 24902.39 | 0.7112 | 0.001*** | 1.000 | 0.7329 |
| Full F~clim. \| (struct. + geog.) | 0.00 | 0.0000 | - | 0.000 | 0.0000 |
| Full F~soil \| (clim. + geog.) | 0.00 | 0.0000 | - | 0.000 | 0.0000 |
| Full F~geog. \| (clim. + soil) | 0.00 | 0.0000 | - | 0.000 | 0.0000 |
| Confounded clim. + soil + geog. | 24902.39 |  |  | 1.000 | 0.7329 |
| Total unconstrained | 8922.00 |  |  |  | 0.2626 |
| Total inertia | 33978.00 |  |  |  | 1.0000 |

Table S5 Summary of genotype-environment association (GEA) SNPs detected from latent factor mixed model (LFMM) and RDA analyses across all 27 populations.

| **SNPs ID** | **LFMM** | **RDA** |
| --- | --- | --- |
| 44503 | bio2 | bio5 |
| 56633 | TAXNWRB | TAXNWRB |
| 71544 | TAXNWRB | TAXNWRB |
| 106388 | sand | sand |
| 193109 | TAXNWRB | TAXNWRB |
| 211051 | TAXNWRB | TAXNWRB |
| 242655 | bio2、bio15 | bio2 |
| 389447 | TAXNWRB | TAXNWRB |
| 399196 | bio2 | bio5 |
| 448212 | sand | sand |
| 470313 | bio2、bio15 | PH_H2O |
| 534730 | bio2、bio15 | bio2 |
| 7719130 | bio2 | TAXNWRB |

Table S6 High-quality BLAST matches obtained in comparison with RAD-sequencing loci against transcriptome of *Adenocaulon himalaicum* and then against *Arabidopsis thaliana* database.

| **SNPs** | **E-value against transcripts** | **Genes ID in *Arabidopsis thaliana*** | **Genes name** |
| --- | --- | --- | --- |
| 56633 | 4e-14 | AT1G78950 | beta-amyrin synthase: BAS |
| 193109 | 6e-05 | AT2G32560 | clock- regulated, F-box with a long hypocotyl 1: CFH1 |
| 211051 | 3e-53 | AT5G23450 | Long-chain base kinase 1: LCBK1 |
| 242655 | 5e-31 | AT3G14570 | glucan synthase-like 4: GSL4 |
| 389447 | 4e-39 | AT5G16980 | Zinc-binding dehydrogenase family proteins: ZDs |
| 399196 | 1e-139 | AT5G56000 | heat shock protein 81.4: Hsp81.4 |

Fig. S1 The FASTSIMCOAL v2.8 scenarios for testing the existence of gene flow between three genetic groups in *Adenocaulon himalaicum*. For each pair group, we investigated the possible models including no geneflow, early geneflow (gene flow right after the split and then no gene flow anymore), recent geneflow (no gene flow initially after the split but gene flow in recent times), different gene flow matrices (higher or lower gene flow right after the split than recently) and constant gene flow (same gene flow strengths since the split until now). The gene flow between group SC and JA was not considered in these current models according to Admixture results.


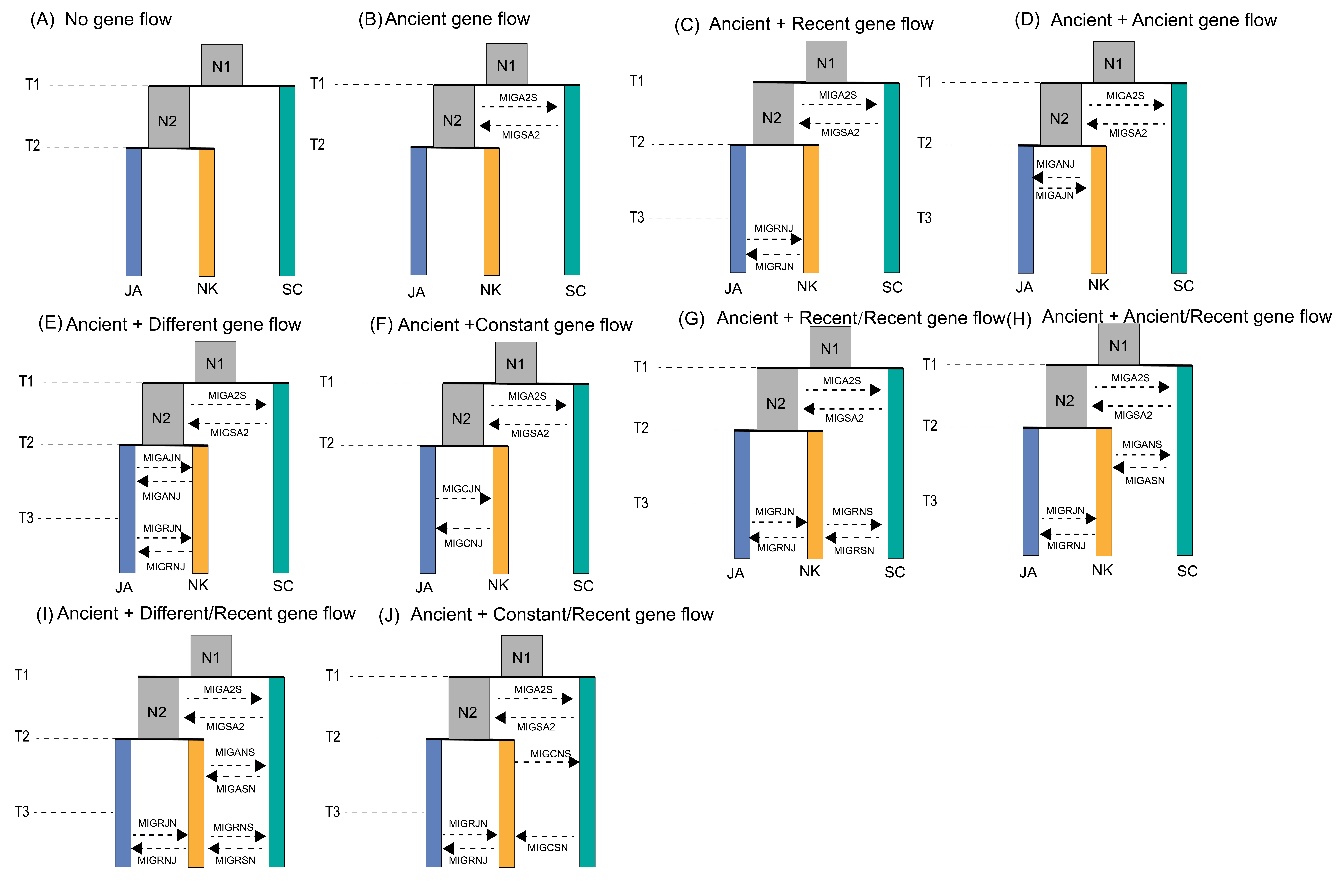


Fig. S2 FASTSIMCOAL v2.8 scenarios for testing the effective population size changes of three genetic groups. For each group, we investigated the possible models including constant contraction, constant expansion, expansion-contraction, contraction-expansion, expansion-expansion and contraction-contraction.


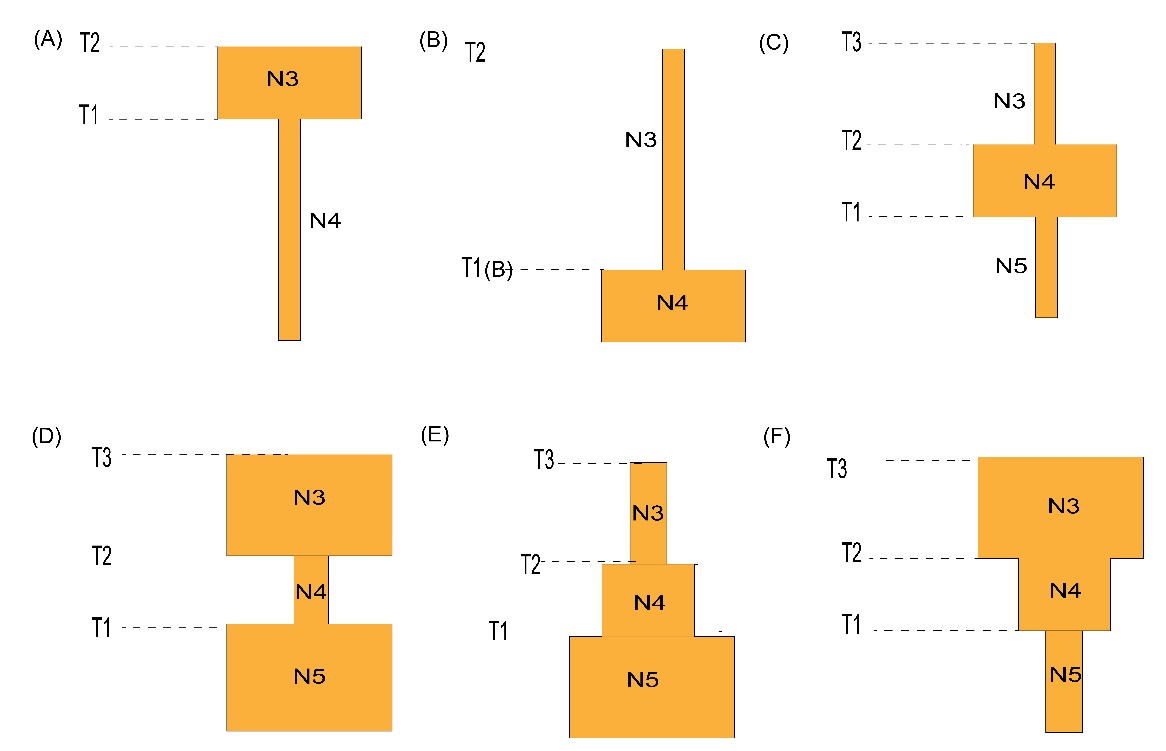


Fig. S3 Distribution of mean coverage of clean reads across individuals for the data set of *Adenocaulon* *himalaicum*.


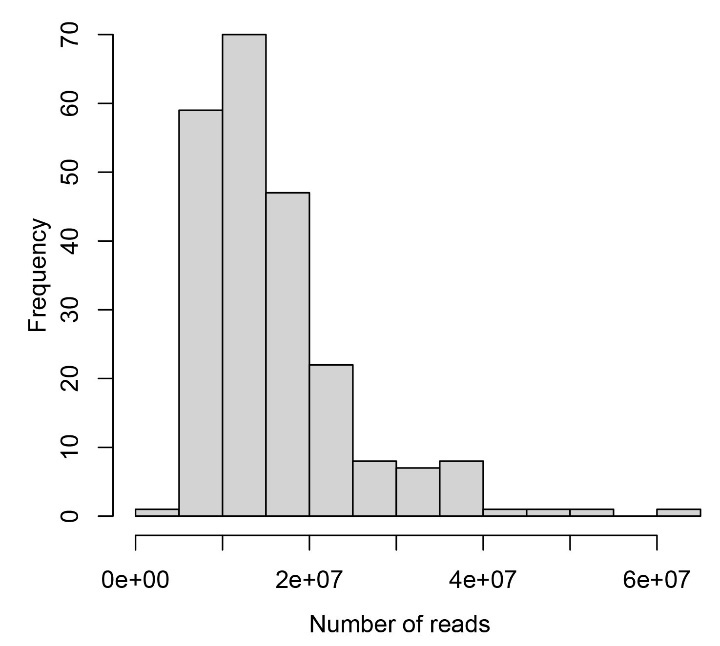


Fig. S4 Distribution of (A) the number of SNPs across individuals and (B) average locus depth of coverage per individual of *Adenocaulon* *himalaicum.*


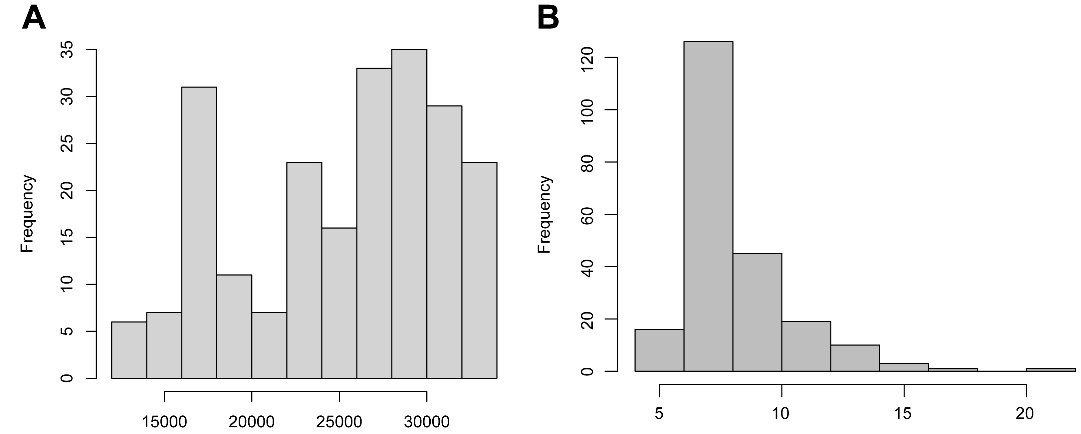


Fig. S5 Venn diagrams showing *F*_ST_ outlier SNPs putatively under divergent selection detected in *Adenocaulon* *himalaicum* based on BayeScan, fdist2 and PCADAPT. The number of unique and common SNPs from each method was given in each area.


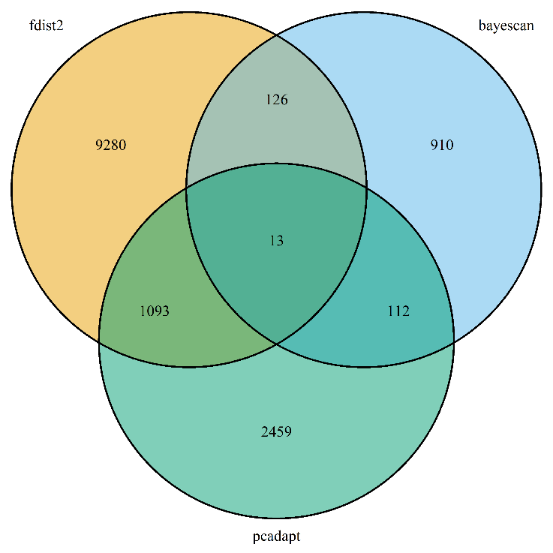


Fig. S6 Population genetic structure of *Adenocaulon himalaicum*. (A) ML-based phylogenetic tree based on neutral SNPs; (B) PCA plot of genetic variation from 27 populations based on neutral SNPs. The variance explained by PCA 1 and PCA2 are 35.27 % and 11.07 %, respectively; (C) a plot of the cross-validation values from K=1 to 15 and (D) genetic structure from K=3 in Admixture based on neutral SNPs. The length of each colored segment represents the proportion of the individual’s genome from K=1 to 15 ancestral genetic groups. The three colored grouped (SC, NK, JA) indicated the three genetic clusters.


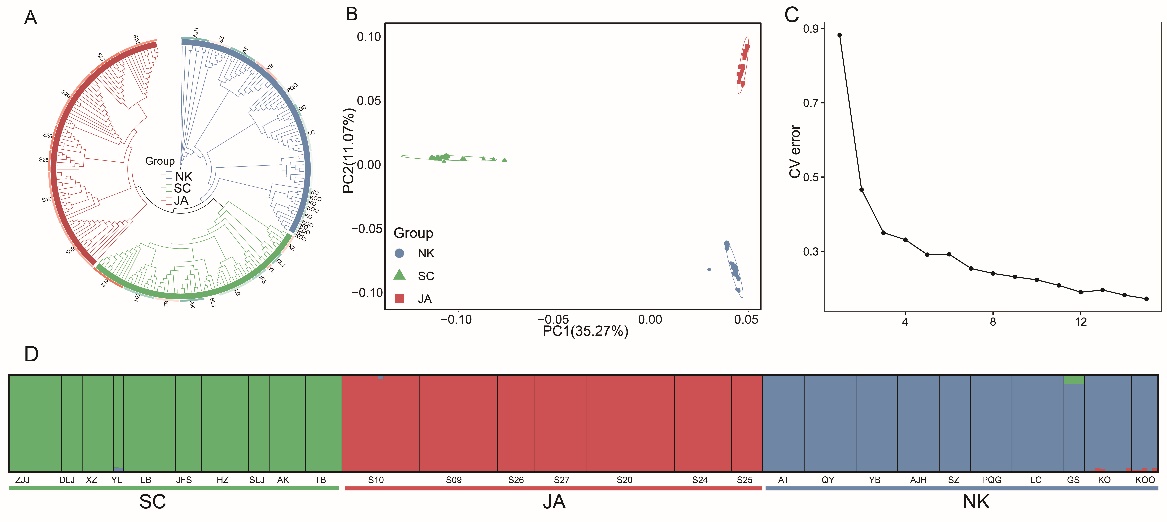


Fig. S7 Comparisons of three summary statistics of three main genetic groups (SC, NK and JA), including (A) nucleotide diversity (Pi); (B) observed heterozygosity (Obs_Het); (C) expected heterozygosity (Exp_Het), and (D) pairwise *F*_ST_ between three groups.


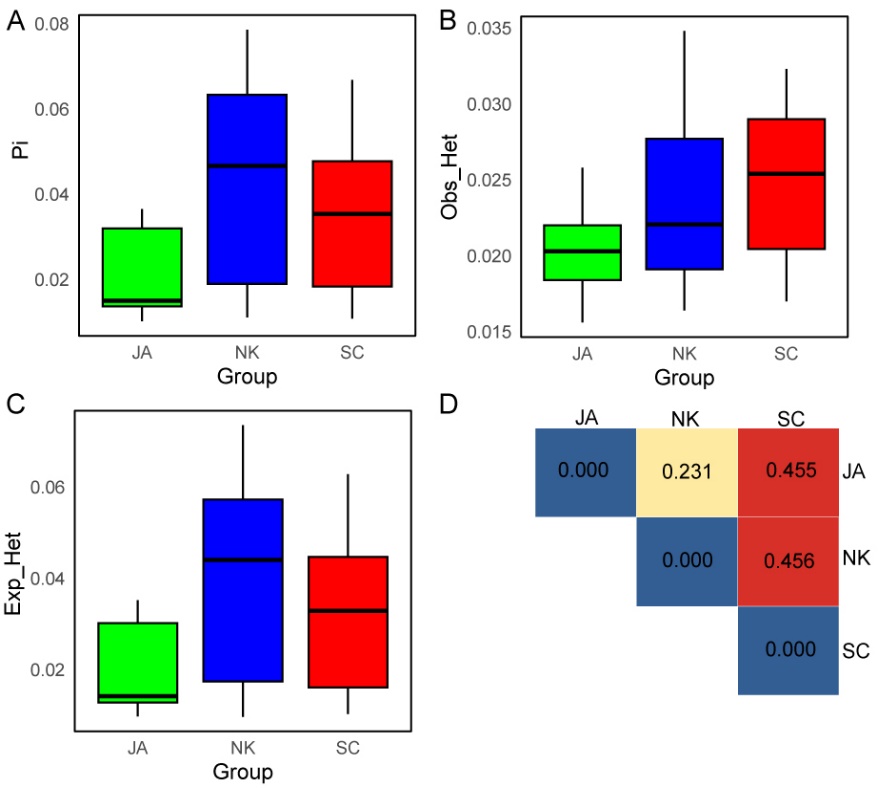


Fig. S8 Heatmap of pairwise genetic differentiation between 27 populations in *Adenocaulon* *himalaicum.*


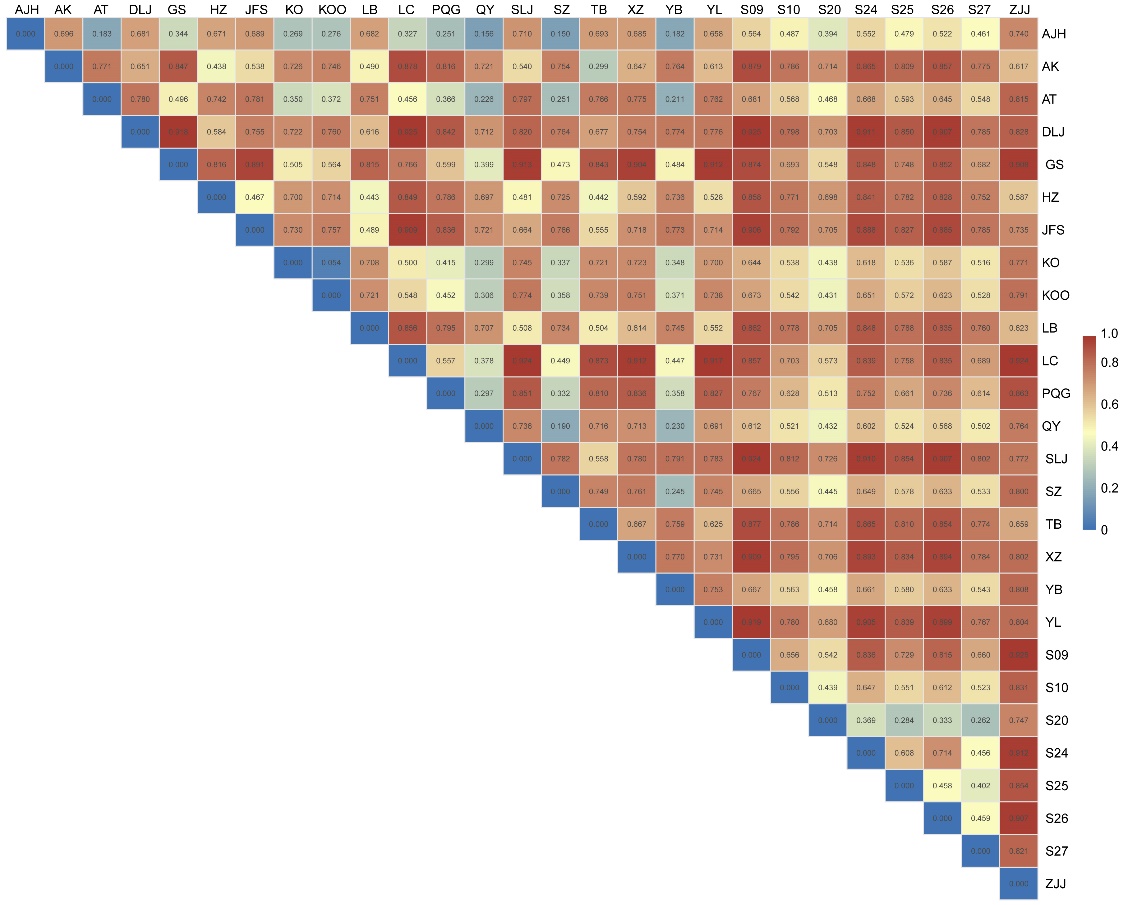


Fig. S9 Relationship between latitude/longitude and genetic diversity measures. Pi: nucleotide diversity; Obs_Het: observed heterozygosity; Exp_Het: expected heterozygosity for *Adenocaulon* *himalaicum* based on neutral SNPs.


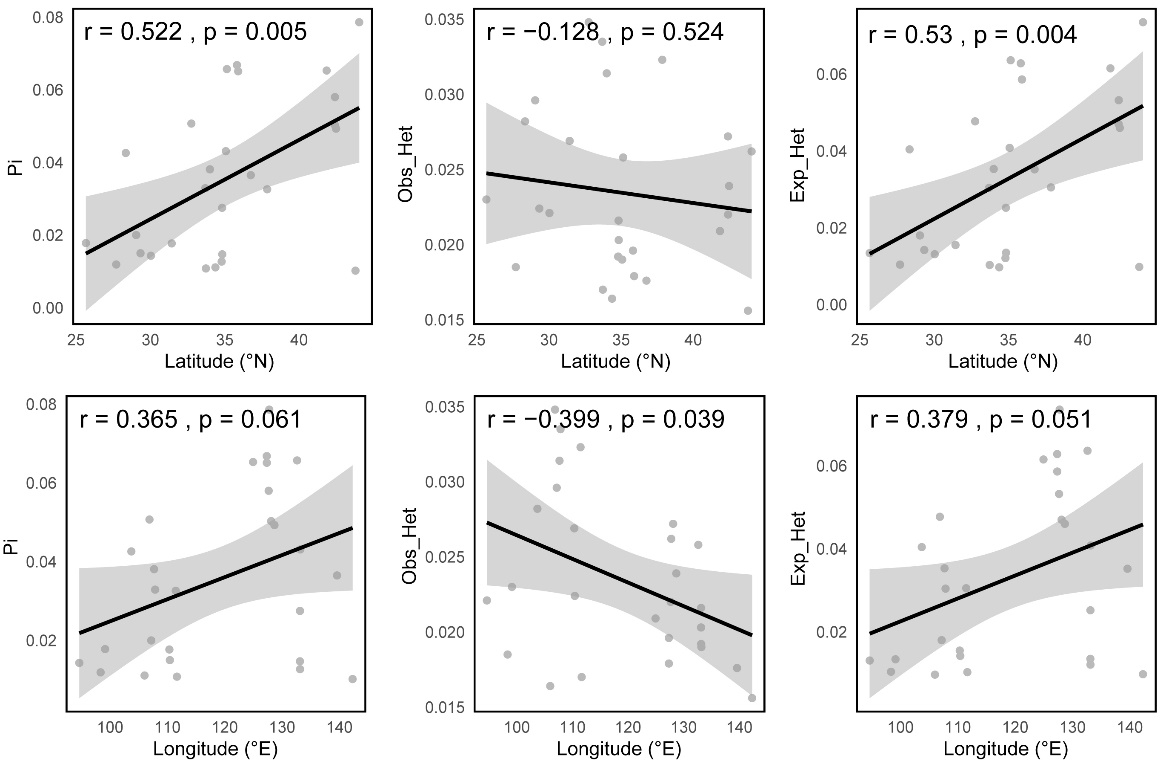


Fig. S10 R^2^-weighted importance of environmental variables that explain genetic gradients for all populations, group south, group north and group JA from gradient forest analysis.


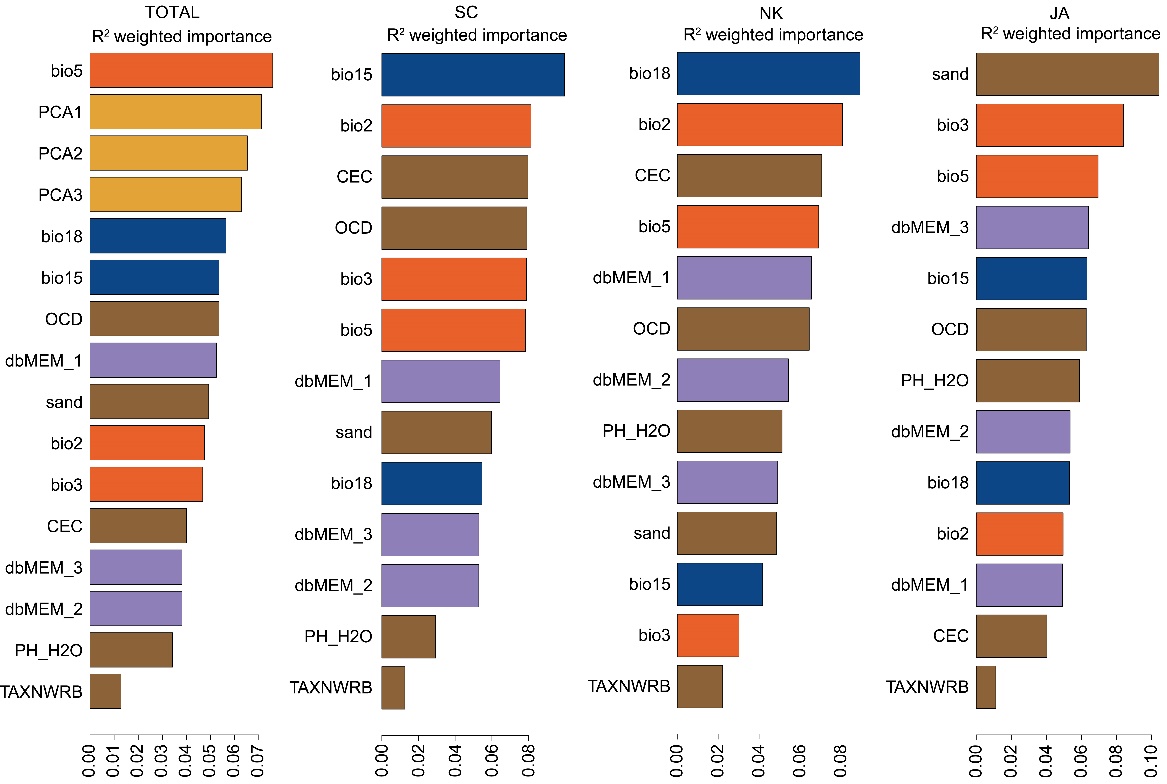


Fig. S11 Cumulative importance of allelic change along the 19 environmental gradients for all populations from neutral SNPs.


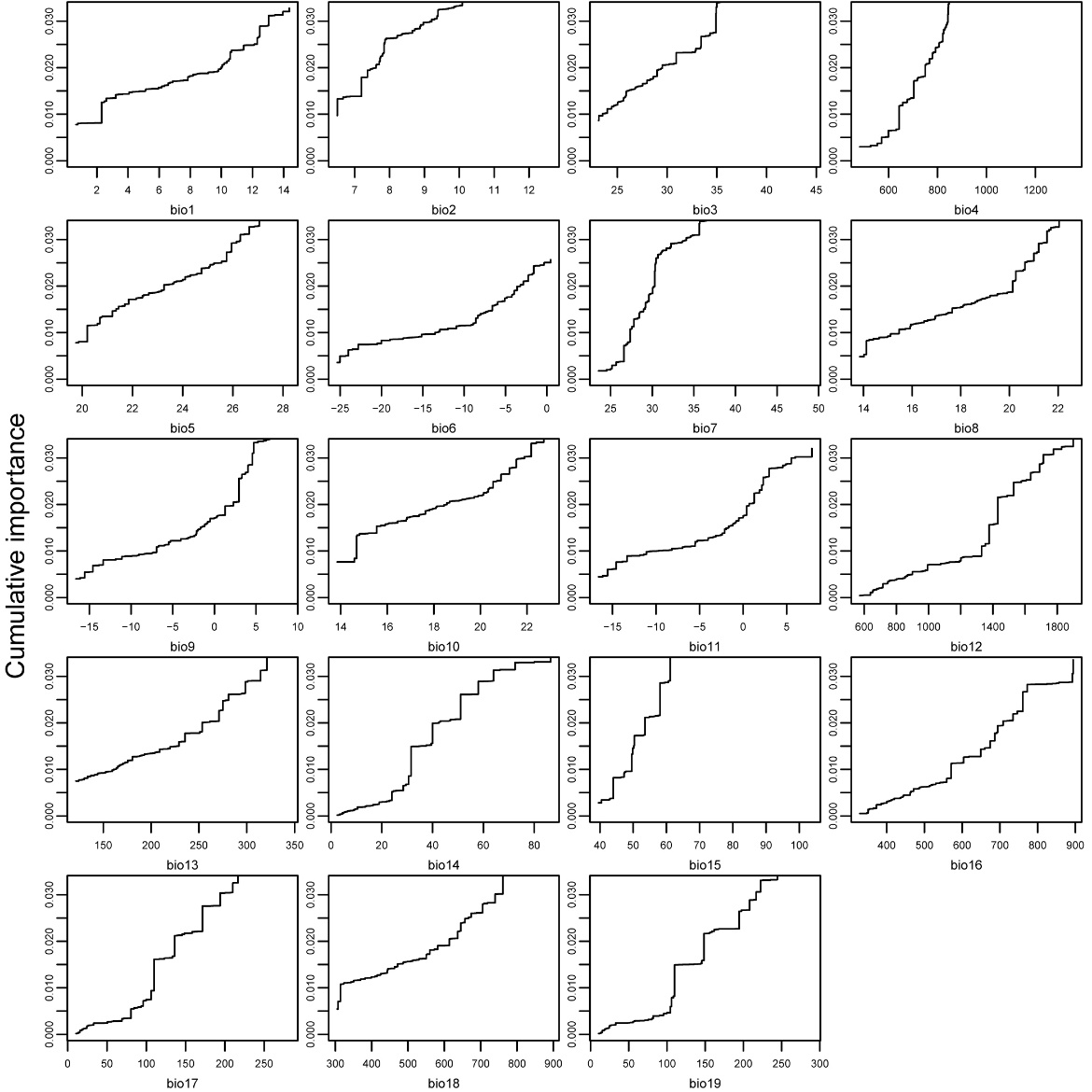


Fig. S12 Venn diagrams showing genotype–environment association (GEA) SNPs in *Adenocaulon* *himalaicum* based on latent factor mixed model (LFMM) and RDA. The number of unique and common SNPs from each method was given in each area. (A) all populations; (B) group JA; (C) group NK; (D) group SC.


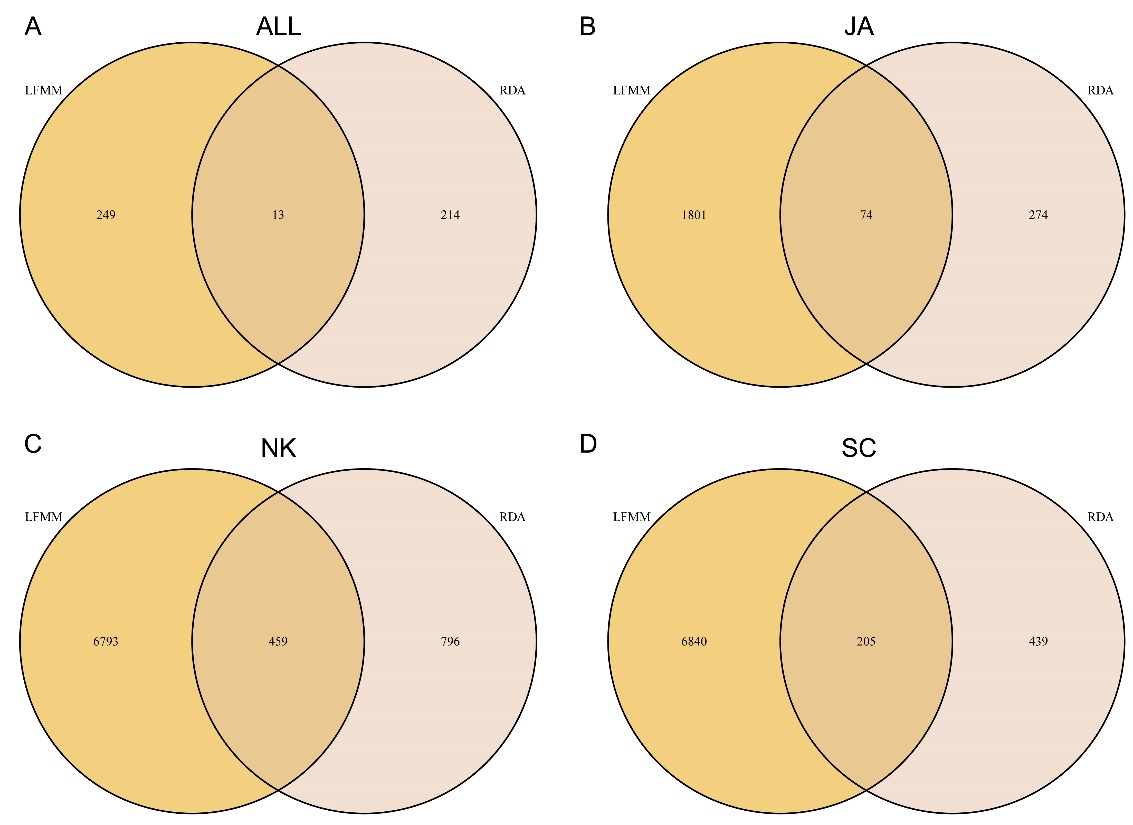


Fig. S13 Prediction of forward genetic offset to future climate change based on 19 environmental variables and all SNPs for under different scenarios. Forward offset under (A) SSP126 and (C) SSP585 during 2041 to 2060; and (B) SSP126 and (D) SSP585 during 2061 to 2080 throughout the range of *Adenocaulon* *himalaicum*.


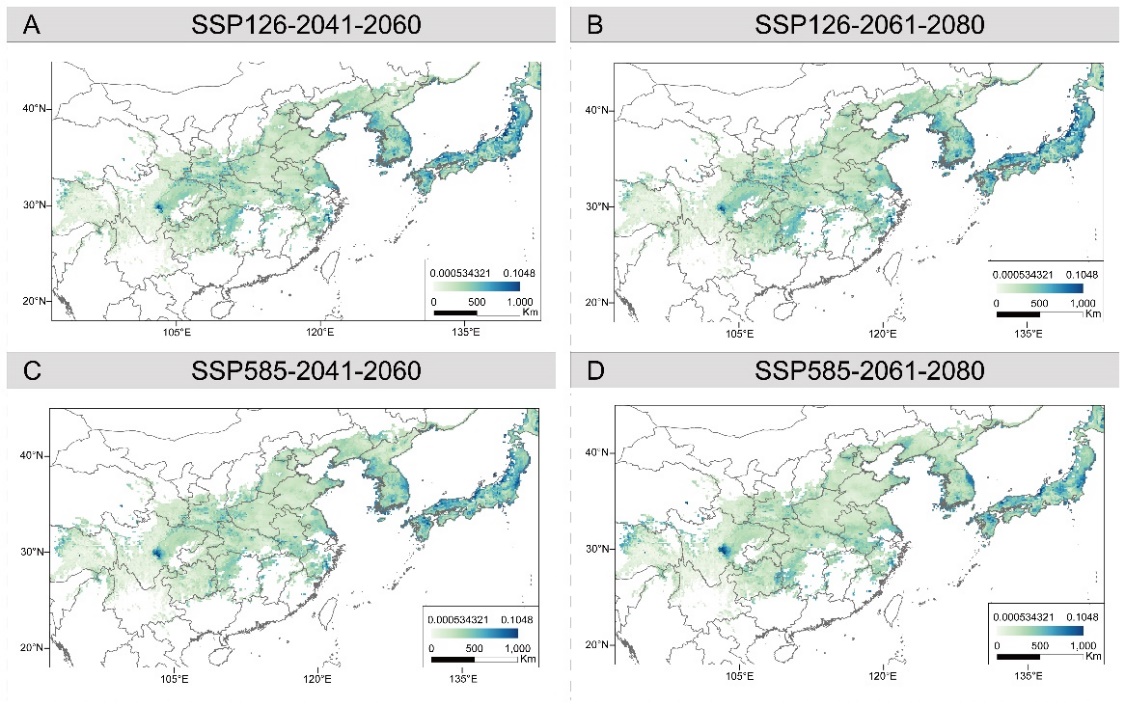


Fig. S14 Prediction of reverse genetic offset to future climate change based on 19 environment variables and all SNPs for under different scenarios. Reverse offset under (A) SSP126 and (C) SSP585 during 2041 to 2060; and (B) SSP126 and (D) SSP585 during 2061 to 2080 throughout the range of *Adenocaulon* *himalaicum*.


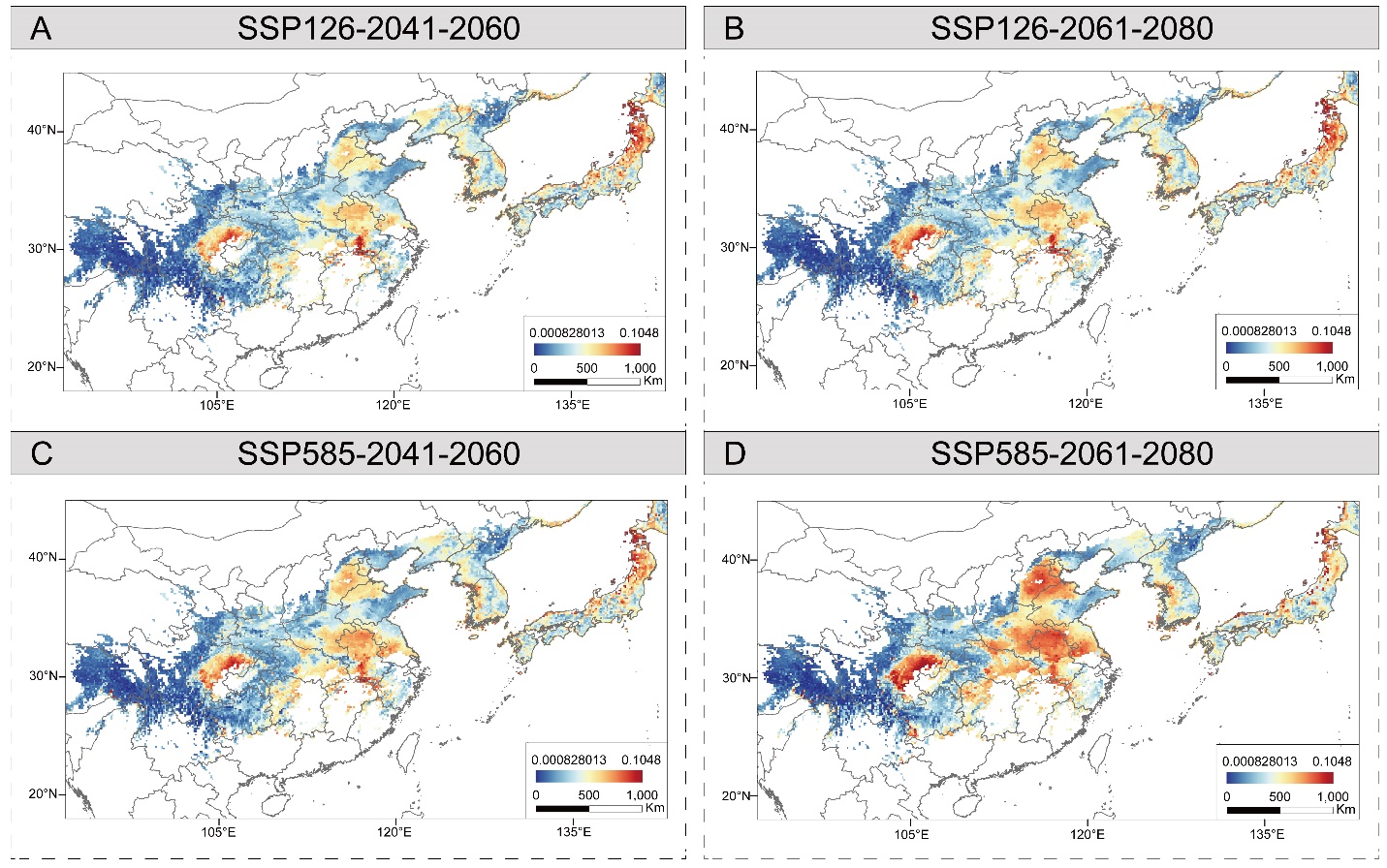


Fig. S15 Prediction of genetic offset to future climate change based on 19 environment variables and 13 core adaptive SNPs for under different scenarios. Local genetic offset under (A) SSP126 and (C) SSP585 during 2041 to 2060; (B) SSP126 and (D) SSP585 during 2061 to 2080 throughout the range of *Adenocaulon* *himalaicum*.


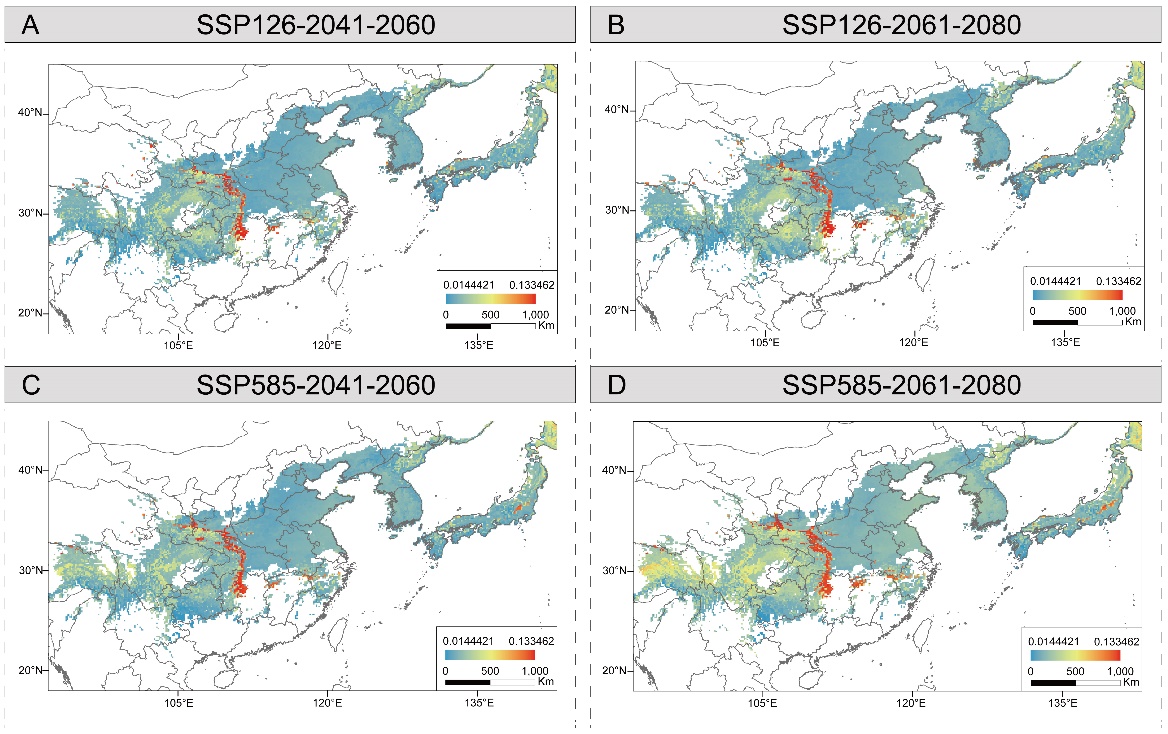


Fig. S16 Prediction of forward genetic offset to future climate change based on 19 environment variables and 13 core adaptive SNPs for under different scenarios. Forward offset under (A) SSP126 and (C) SSP585 during 2041 to 2060; and (B) SSP126 and (D) SSP585 during 2061 to 2080 throughout the range of *Adenocaulon* *himalaicum*.


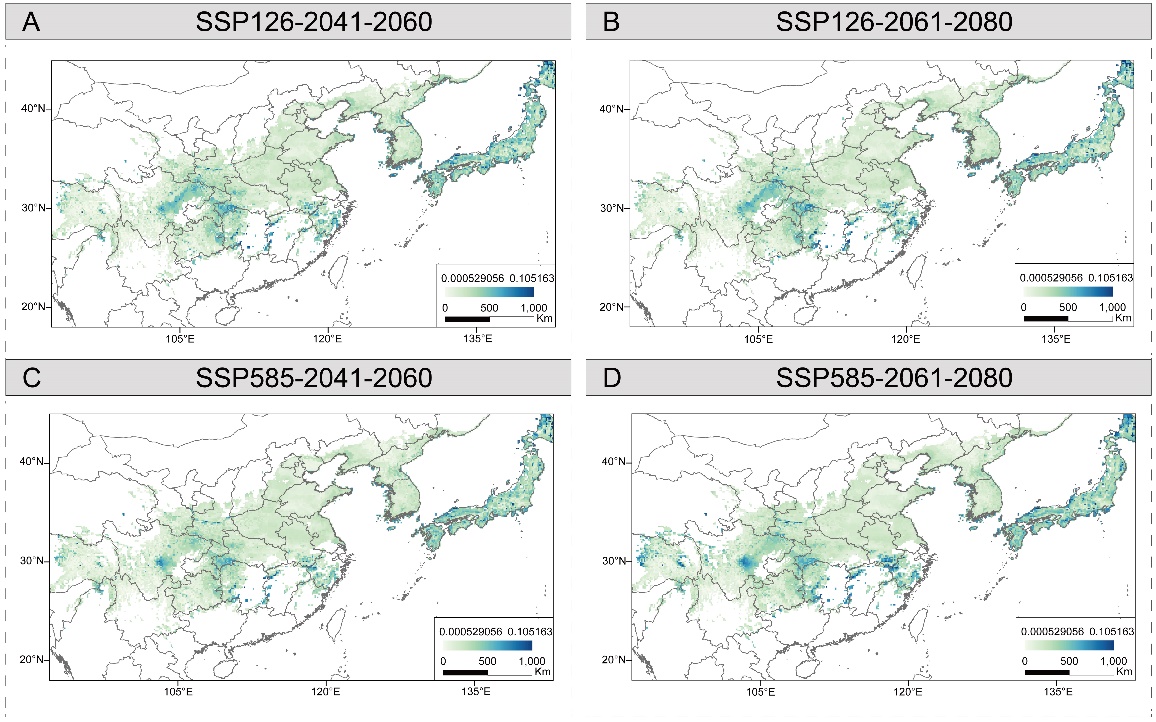


Fig. S17 Prediction of reverse genetic offset to future climate change based on 19 environment variables and 13 core adaptive SNPs for under different scenarios. Reverse offset under (A) SSP126 and (C) SSP585 during 2041 to 2060 and (B) SSP126 and (D) SSP585 during 2061 to 2080 throughout the range of *Adenocaulon* *himalaicum*.


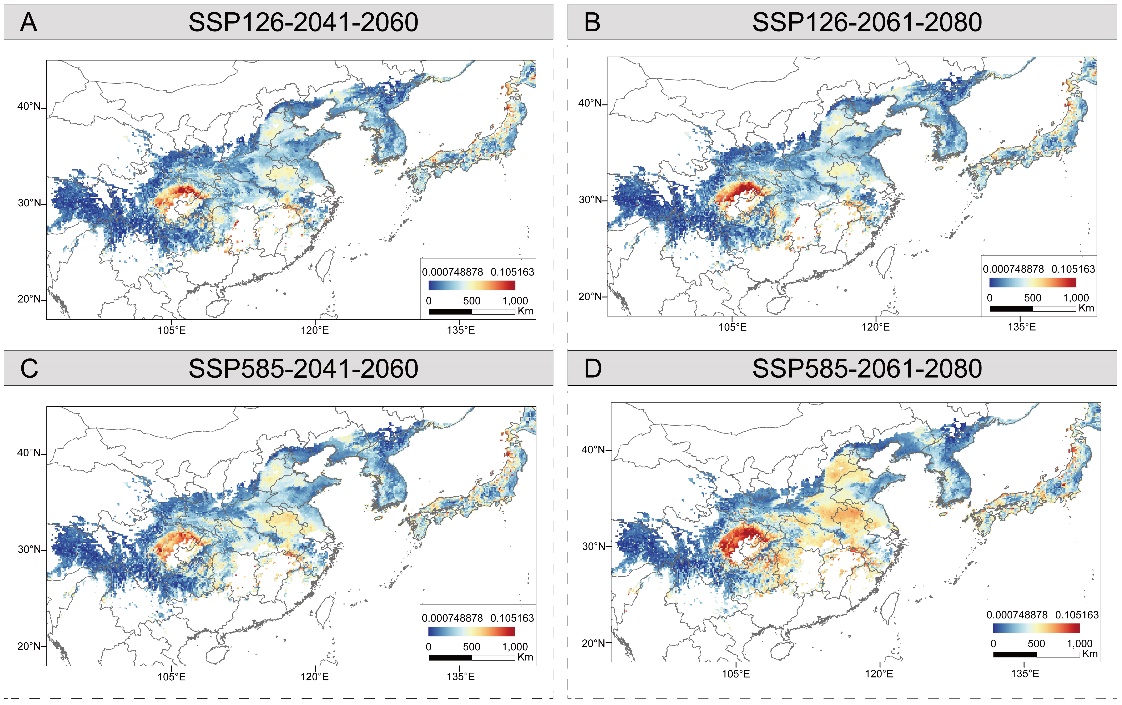


Fig. S18 Prediction of RGB genetic offset to future climate change based on 19 environment variables and 13 core adaptive SNPs for under different scenarios. RGB map of local (red), forward (green), and reverse (blue) under (A) SSP126 and (C) SSP585 during 2041 to 2060; and (B) SSP126 and (D) SSP585 during 2061 to 2080 throughout the range of *Adenocaulon* *himalaicum*.


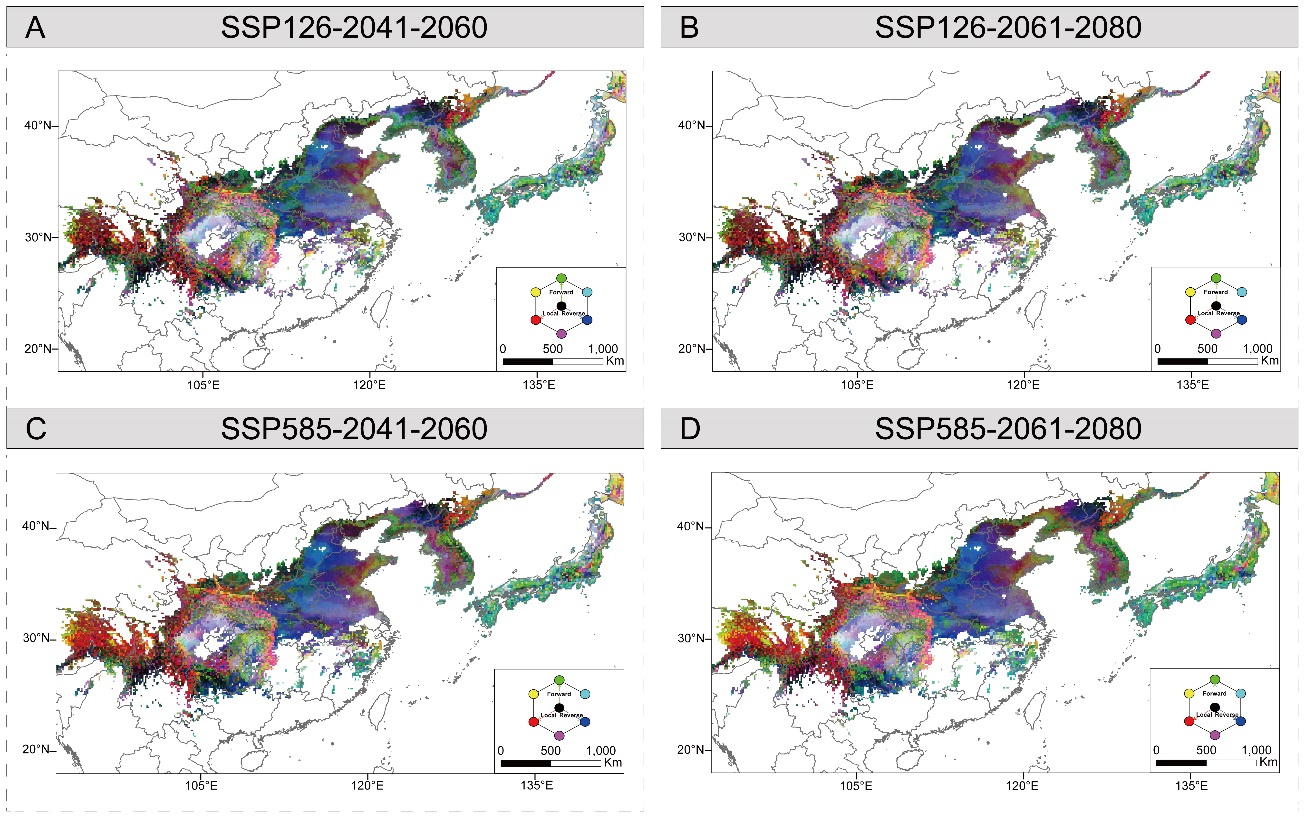


Fig. S19 Correlations between environment variables and genetic offset under different future climate change of (A-B) SSP126 and (C-D) SSP585 during 2041 to 2060 and 2061 to 2080 of *Adenocaulon* *himalaicum*.


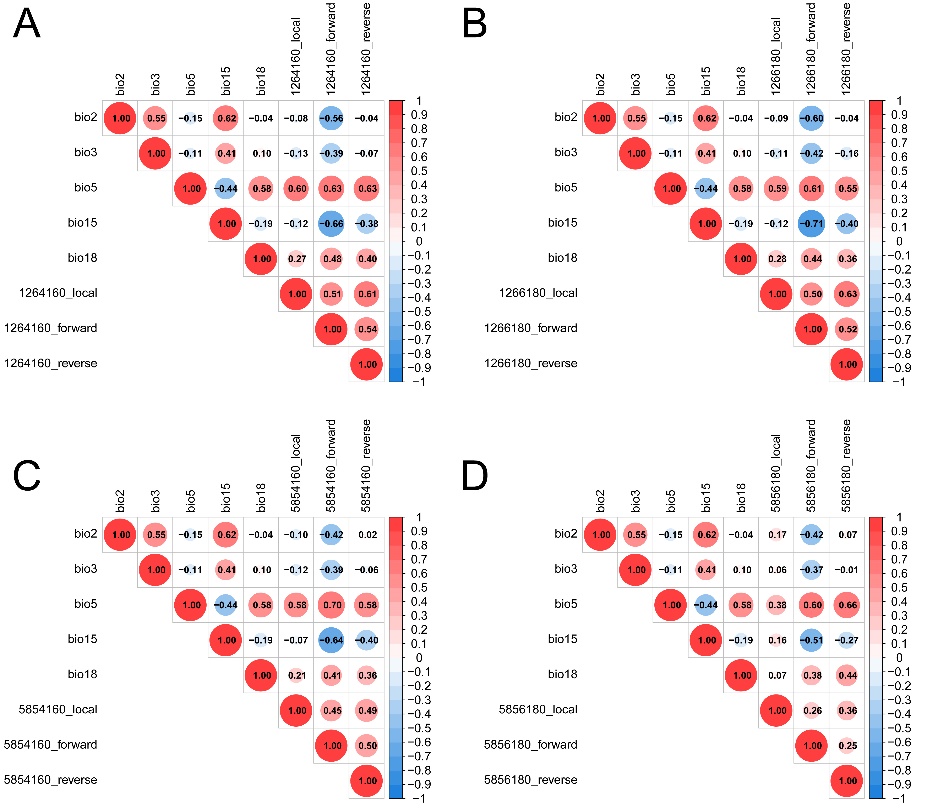

Supplement: Supplementary file 1 — Data S1: mec70068‐sup‐0001‐Supinfo01.docx. [file MEC-34-e70068-s001.docx]
